# Delay models for the early embryonic cell cycle oscillator

**DOI:** 10.1371/journal.pone.0194769

**Published:** 2018-03-26

**Authors:** Jan Rombouts, Alexandra Vandervelde, Lendert Gelens

**Affiliations:** Laboratory of Dynamics in Biological Systems, Department of Cellular and Molecular Medicine, University of Leuven, 3000 Leuven, Belgium; University of California Irvine, UNITED STATES

## Abstract

Time delays are known to play a crucial role in generating biological oscillations. The early embryonic cell cycle in the frog *Xenopus laevis* is one such example. Although various mathematical models of this oscillating system exist, it is not clear how to best model the required time delay. Here, we study a simple cell cycle model that produces oscillations due to the presence of an ultrasensitive, time-delayed negative feedback loop. We implement the time delay in three qualitatively different ways, using a fixed time delay, a distribution of time delays, and a delay that is state-dependent. We analyze the dynamics in all cases, and we use experimental observations to interpret our results and put constraints on unknown parameters. In doing so, we find that different implementations of the time delay can have a large impact on the resulting oscillations.

## Introduction

**Fig 1:**
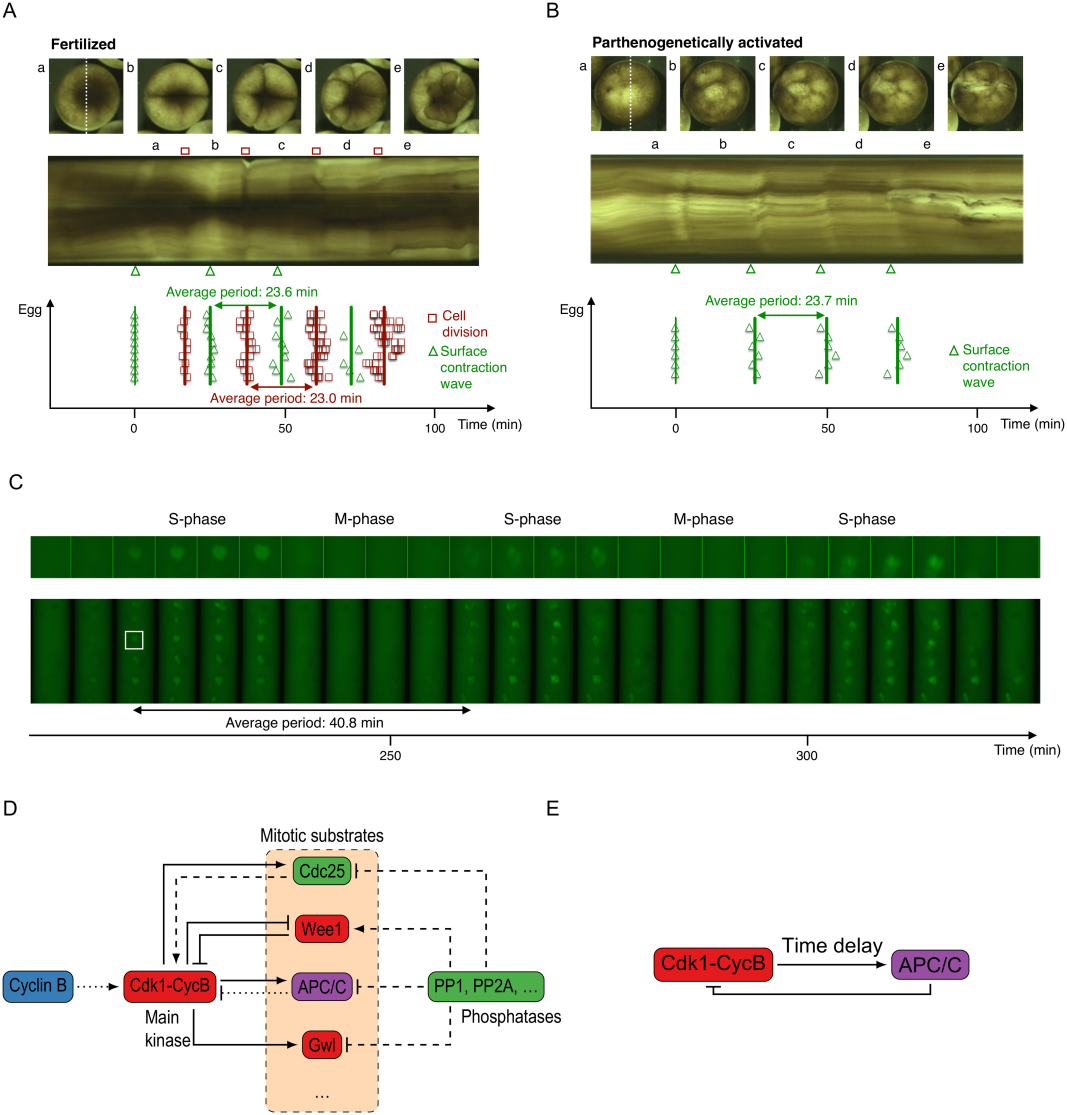
The underlying network of interacting proteins generates periodic cell cycle oscillations in the *Xenopus* embryo. A and B) Timing of surface contraction waves for fertilized (A) and parthenogenetically activated (B) *Xenopus* eggs, and cell division timing for fertilized eggs. Surface contraction waves are indicated by green triangles, cell division timings by red squares. Time is expressed relative to the timing of the first surface contraction wave. A) Top: images of a fertilized egg in the one cell (a), two cell (b), four cell (c), eight cell (d) and sixteen cell stage (e). Middle: kymograph of this fertilized egg: the intensity along the dotted line in (a) is plotted as a function of time. Bottom: timings of the cell divisions and surface contraction waves for ten fertilized eggs. Full lines indicate the average timing of these events. B) Top: images of a parthenogenetically activated egg. Middle: kymograph of this egg. Bottom: timing of the surface contraction waves for six parthenogenetically activated eggs. C) Images of nuclear envelope breakdown and reformation in a cycling extract prepared from *Xenopus* eggs, supplemented with demembranated sperm nuclei and GFP-NLS. The extract was visualized in a Teflon tubing with an inner diameter of 300 μm and a length of approximately 10 mm, submerged in mineral oil. Time is expressed relative to the point when the extract was warmed to 24 °C. D) Schematic view of some of the proteins that regulate the cell cycle. Cdk1-cyclin B is the main kinase that phosphorylates substrates in mitosis. Kinases are red, phosphatates are indicated in green. Full lines indicate phosphorylation, dashed lines indicate dephosphorylation. The symbol → means activation and ⊣ means inhibition. E) Simplified model: only the core oscillator is retained, the interactions are summarized by including high ultrasensitivity and a time delay in the response of APC/C.

The cell cycle is one of the most fundamental processes in living organisms. In order to survive and grow, a cell needs to proceed in a well-controlled fashion through DNA replication, mitosis and growth. The cell cycle is tightly regulated, since a failure in this machinery can lead to diseases such as cancer. The early embryonic cell cycle of the frog *Xenopus laevis* has been used as a model system to understand the biochemical network that underlies the cycling behavior. This embryonic cell cycle can be seen as an autonomous biochemical oscillator. After the first cycle, which takes about 80 minutes, cycles 2–12 are regular and fast [1]. Each cycle then only takes about 25 minutes each (Fig 1A), where the cells switch between S phase and M phase, without any gap phases or checkpoints in between. These regular oscillations even persist with exactly the same period when parthenogenetically activated (Fig 1B). In this case no actual cell divisions occur, but the biochemical oscillations continue as can be seen by so-called surface contraction waves (SCWs) appearing with the same periodicity (Fig 1B). Such SCWs are changes in the pigmentation of the egg cortex that occur before each cell divides [2], and it is believed that these SCWs are associated to waves traveling through the egg, thus triggering the cell to divide [3, 4]. The fact that these early embryonic oscillations are so regular, and occur in the absence of checkpoints and fertilization, makes this cell cycle more amenable to detailed study, both in the lab and using mathematical models. This idea is further strengthened by the fact that one can even pool thousands of frog eggs into one cytoplasmic cycling egg extract [5], and even then the biochemical oscillations can persist *in vitro*, as shown in Fig 1C by the fluorescent nuclei that periodically appear (S phase) and disappear (M phase). In this case, however, the period of the oscillations is different, and it remains unclear why.

The possibility to make frog egg cell extracts and reconstitute transitions between M phase and S phase *in vitro* has allowed researchers to dissect the underlying network of interacting proteins that lies at the heart of cell cycle oscillations (Fig 1D). The main mitotic regulator has been identified as Cdk1 (cyclin-dependent kinase 1), which binds to cyclin B, a protein that is produced at a constant rate. When the Cdk1-cyclin B complex is in its active phosphorylation state, it goes on to phosphorylate many mitotic substrates that trigger mitosis. In order to exit M phase, Cdk1 activates an E3 ubiquitin ligase called APC/C (anaphase-promoting complex / cyclosome), which tags cyclin B for degradation by the proteasome [6]. As such, the Cdk1-cyclin B complex disassembles. Of course, upon mitotic exit, all of the substrates then need to get dephosphorylated by a number of counteracting phosphatases, which are highly regulated as well [7, 8].

Fig 1D shows this basic biochemical scheme and highlights that various interactions exist between Cdk1 substrates and Cdk1 itself. For example, Cdk1 activates the phosphatase Cdc25, while Cdc25 in turn also activates Cdk1, constituting a positive feedback loop [9]. Cdk1 also inhibits Wee1, a kinase that itself inhibits Cdk1 activity [10, 11]. Such a double-negative feedback loop is known to generate bistability, and indeed this circuit has been found to introduce bistability in the activation of Cdk1 by increasing cyclin B levels [12, 13]. Similar feedback loops might also operate on the level of the main mitotic phosphatases PP1 and PP2A, as motivated by recent results [14–16]. Moreover, experiments have also revealed that the (de)activation of proteins in the cell cycle circuit often occurs in a highly nonlinear fashion, with the response of Wee1, Cdc25 and APC/C to Cdk1 activity all found to be very steep [17–20]. This sigmoidal, switch-like response is called ultrasensitivity. Finally, the activation of APC/C was measured to occur with a time delay [19].

Not all known protein interactions are indicated in Fig 1D, and it is very likely that the picture is even more complex than currently believed. Including all known interactions into a detailed mathematical model for the embryonic cell cycle, therefore, quickly leads to a very large set of equations with a correspondingly large set of parameters. Although many of those parameters have been measured directly, or fitted from experimental data [16–21], large uncertainties still exist. Measurements can depend on the experimental conditions and fitting parameters in large models is a nontrivial task. Different parameter sets can lead to similar model behavior, making it hard to accurately infer parameter values from data [22]. In order to reduce this complexity, we focus on the fast cell cycles 2–12, where Tsai et al. [20] have experimentally demonstrated that the ratio of Cdc25/Wee1 is increased such that Wee1 is no longer able to generate a bistable response of Cdk1 activity vs. cyclin B. Instead, all Cdk1-cyclin B complexes are all quickly converted into their active state by the strong activity of Cdc25. As a result, we can simplify the system by assuming cyclin B levels equal those of active Cdk1-cyclin B, and omit the feedback loops involving Cdc25 and Wee1. What we are left with is a time-delayed negative feedback loop (Fig 1E), a motif known to cause oscillations [23, 24]. Cdk1-cyclin B activates APC/C in an ultrasensitive [19, 20] and time-delayed [19, 25] way, after which APC/C inactivates Cdk1-cyclin B. We assume that any additional feedback loops act to alter either the time delay and/or ultrasensitivity in the response of APC/C to Cdk1 activity.

Although the importance of this time delay has been acknowledged from the beginning [26], the way it is implemented varies from model to model. A time delay can be included in a model by adding extra intermediate variables. Novak and Tyson [27] and Marlovits et al. [21] included an artificial intermediary enzyme in their model to account for the time delay. Pomerening et al. [28] and Tsai et al. [20] do the same but identified this protein as Polo-like kinase 1. One of the models analyzed by Yang and Ferrell [19] introduces not one, but a chain of intermediary variables, which could correspond to different phosphorylation states of APC/C. Multisite phosphorylation is one of the mechanisms that could induce both a time delay and an ultrasensitive response [29, 30]. Another way of including the time delay is by doing so explicitly, which means going from ordinary differential equation (ODE) models to delay differential equation (DDE) models. Although this complicates the mathematics a bit, these models include the time delay in a conceptually clear way. Srividhya and Gopinathan [31] study a DDE model of seven variables for the general eukaryotic cell cycle, mentioning that the time delay is used as a substitute for a cascade of phosphorylation steps, an approach for which they provide some justification in another paper [32]. An explicit delay was also used by Yang and Ferrell [19] next to their model with intermediates. Although a value for the time delay based on experiment was used, the period of the oscillations in the different models did not seem to correspond with the experimental results.

In this work, we aim to shed light on how ultrasensitivity and time delay influence cell cycle oscillations in a simple negative feedback model (Fig 1E). We describe various different ways of including the time delay and analyze the dynamics of the model in each case. Although the model is deceptively simple, we believe it captures the essence of the cell cycle oscillator and we demonstrate that implementing the time delay differently can have a large impact on the resulting oscillations. Initially, we assume the delay is one well-defined time, such that a DDE with a fixed delay time can be used. Next, we extend the analysis to DDE models with distributed delays. Finally, we allow the time delay to be different for APC/C activation and deactivation, introducing a so-called state-dependent time delay. Besides a thorough analysis of the delay models, we provide general messages that can be useful to modelers who want to include a time delay in their models, and we discuss how model parameters can be extracted from the available experimental data. This model is one of the simplest cell cycle models we know of and has not been studied in this form. It is the first cell cycle model in which different delay implementations are explicitly compared to each other, and the first time a state-dependent delay is used to model different activation and inactivation times of APC/C. We explore the effect of time delay in such a simple model because it allows to extract useful, unambiguous messages. Later, more complicated models could be used to see how predictions from this simple model hold up.

## Results

### Ultrasensitivity and time delay are both required for oscillations

**Fig 2:**
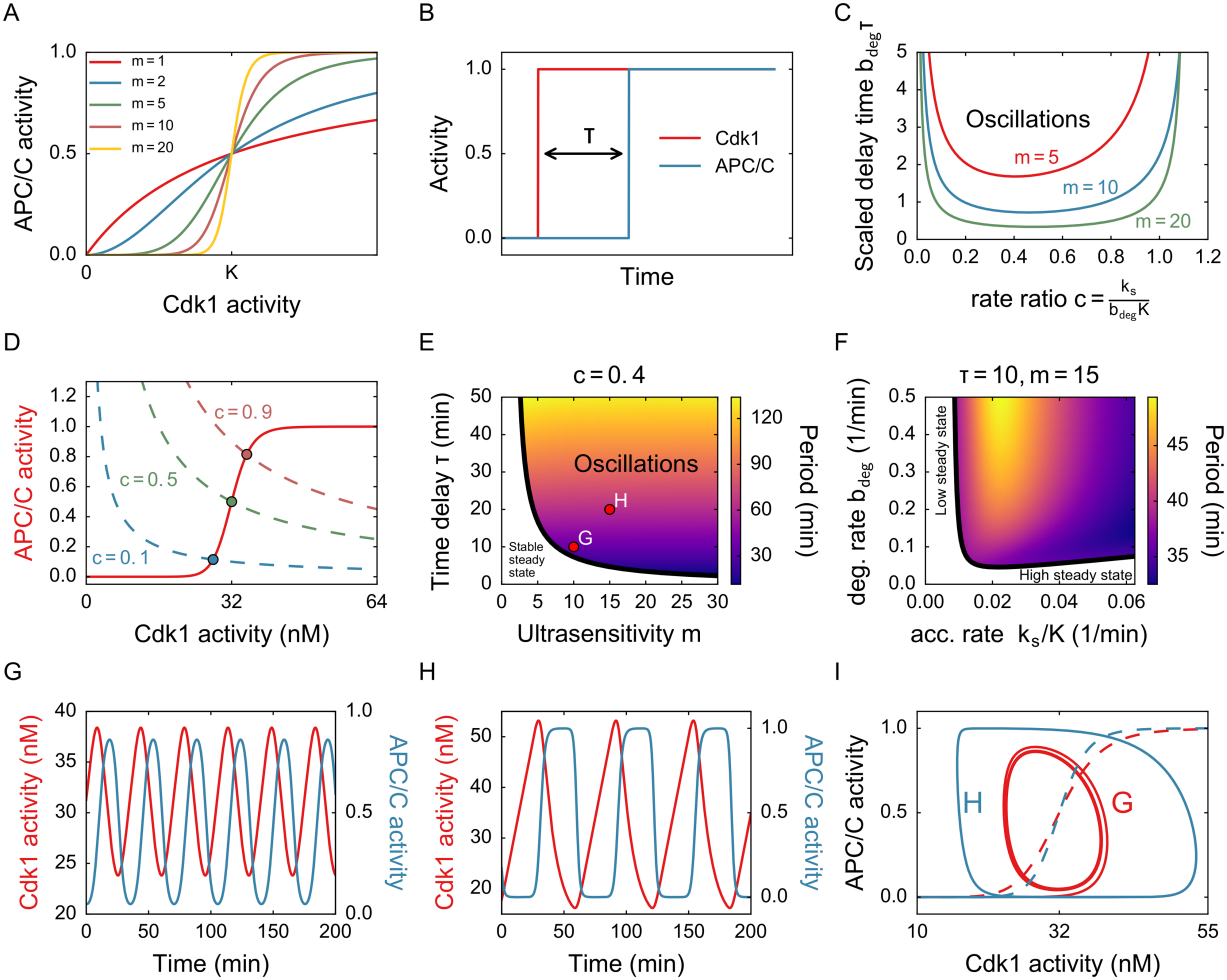
Oscillations exist when the response is steep and the time delay is long enough. A) Steady state response of APC/C to Cdk1 activity. Higher *m* corresponds to a steeper response. B) When Cdk1 is suddenly activated, APC/C follows after a fixed time in the model with one discrete delay. C) Phase diagram for parameters $c=\frac{k_{s}}{b_{\text{deg}}K}$ and *τ*, for different values of *m*. Increasing *m* corresponds to an larger region of oscillations. D) Fixed point location. The dots show the APC/C activity in steady state, for different *c*. The fixed point can be found as the intersection of the APC/C response curve and the dashed lines, which are derived by putting the right hand side of Eq (1) to zero. E) Phase diagram for parameters *m* and *τ*, with period in color. The points correspond to parameter values used for the timeseries in G and H. F) Phase diagram for parameters *k*_*s*_/*K* and *b*_deg_ with period in color. G) Time series (sinusoidal) for *m* and *τ* denoted by point G. H) Time series (relaxation-like) for *m* and *τ* denoted by point H. Other parameters for G and H: *k*_*s*_ = 1.28 nM/min, *b*_deg_ = 0.1 min^−1^. I) The two timeseries from G and H plotted in a plane. Note that APC/C is not an independent variable, but is a time-delayed function of Cdk1. The dashed line denotes the steady-state reponse of APC/C to Cdk1. The oscillations occur around the threshold value.

The basic model illustrated in Fig 1E is phenomenological and it includes the two main observations from the recent paper by Yang and Ferrell [19]: the response of APC/C to Cdk1 activity is ultrasensitive (Fig 2A) and the activation is time delayed (Fig 2B). We assume that cyclin production is constant with rate *k*_*s*_ and that cyclin B binds immediately to Cdk1 to produce an active Cdk1-cyclin B complex. The degradation of cyclin B by APC/C is modeled using mass action, with rate *b*_deg_. This degradation immediately deactivates the Cdk1-cyclin B complex. The activity of APC/C is explicitly given as a time-delayed ultrasensitive function of Cdk1. We choose a Hill function with exponent *m* and threshold value *K* to model this ultrasensitivity. Yang and Ferrell [19] fitted an exponent of about 17, which corresponds to a very sharp response, and they measured a time delay of about 15 minutes. This results in the following DDE model:

$$\begin{array}{c} \frac{d[\text{Cdk1}]}{dt}=k_{s}-b_{\text{deg}}\left[\text{Cdk1}\right]\frac{{\left[\text{Cdk1}\right]}^{m}\left(t-\tau \right)}{K^{m}+{\left[\text{Cdk1}\right]}^{m}\left(t-\tau \right)}, \end{array}$$ (1)

where the final term denotes the activity of APC/C. A list of parameter values and biologically realistic ranges can be found in Table 1. All values are based on the papers by Tsai et al. [20] and Yang and Ferrell [19]. The range for studied time delays is larger than strictly biologically plausible.

**Table 1:** Parameter meaning and values for the model in Eq (1).

Parameter	Meaning	Typical Value/Range
*k*_*s*_	Cyclin production rate	1-1.5 nM/min
*b*_deg_	Cyclin degradation rate	0.04-0.4 min^−1^
*K*	Threshold for APC/C activation	32 nM
*m*	Hill exponent	>15
*τ*	Delay time	15 min (5–40 min (?))

Our equation is an example of a time-delayed negative feedback loop, which bears resemblance to other oscillating systems in biology. One of these systems is the Goodwin oscillator, which can be considered as a prototype of a nonlinear delayed feedback oscillator [33]. Goodwin’s oscillator was originally used to model the repression of a gene by its own protein product and has been used to model the circadian clock [34, 35] and somitogenesis [36]. In the original model the time delay was implemented by adding two intermediate variables, but models with an arbitrary number of variables [37] and variants with an explicit time delay [38] have been studied too. The nonlinearity in the Goodwin model is represented by a Hill function with a high value of the exponent, which is similar to the case in our model. This high value has been criticized as being unrealistic, although some mechanistic explanations have been put forward [39]. Some of the results of our model correspond to features also found in the Goodwin model, and will likely apply to biological oscillators in general.

Analyzing the stability of this simple cell cycle model 1 shows that it relaxes to a unique steady state, unless the delay time is large enough (details of the analysis can be found in S1 Text). This behavior is typical in time-delayed negative feedback loops [23]: long time delays facilitate oscillations. The critical time delay *τ*_*c*_ at which the system starts oscillating depends on the other parameters. There is a clear tradeoff between the required ultrasensitivity and delay time: very steep responses need only little delay to produce oscillations (Fig 2C and 2E), whereas for *m* too low the system never oscillates. For *m* = 1, a hyperbolic response, it can be shown mathematically that the system always ends up in the steady state. This has been observed in other systems as well, such as the Goodwin oscillator [40].

The ratio between the normalized cyclin accumulation rate *k*_*s*_/*K* and its degradation rate *b*_deg_ is a dimensionless parameter which we will call *c*:

$$\begin{array}{c} c=\frac{k_{s}}{b_{\text{deg}}K}. \end{array}$$ (2)

This value influences the behavior of the system significantly (Fig 2C). When *c* is too low or too high, oscillations only occur for very large delay times. An intermediate value of *c* ≈ 1/2 is most favorable for oscillations. This corresponds to *b*_deg_ ≈ 2*k*_*s*_/*K*, or a degradation rate which is approximately twice the accumulation rate, normalized with respect to the threshold concentration. Note that the value of *c* also determines the steady state of the system. Recall that for smaller time delays, the system ends up in this steady state, but for higher time delays this state becomes unstable and the system starts oscillating around the steady state (S1 Fig). Low *c* entails a steady state with low APC/C activity (Fig 2D). The results from the phase diagram in Fig 2C can then be interpreted as follows: when the steady state is very low or very high, a longer time delay is needed to destabilize it and for oscillations to occur.

The period of the oscillations does not depend on all parameters. The delay time is very important, but the value of *m*, which determines the steepness of the response, has little influence on the period of the oscillation (Fig 2E). Accumulation and degradation rates are also important (Fig 2F): higher degradation rates generally lead to longer periods, whereas there is an intermediate accumulation rate for which the period is largest. Shape and amplitude of the oscillations depend on how close to the phase boundary the parameters lie: close to the boundary, oscillations are small in amplitude and sinusoidal (Fig 2G). Farther away, they obtain a sawtooth-like character (Fig 2H). The detailed dependence of amplitude on the parameters can be found in S2 and S3 Figs.

### For high ultrasensitivity, the oscillation period can be found analytically

As mentioned before, the response of APC/C to active Cdk1 has been experimentally found to be very steep (*m* ≈ 17 in [19]). Moreover, we noticed that the oscillation period depends little on the value of *m* (Fig 2E). This motivated us to study the limit for *m* → ∞, which greatly simplifies the equation and allows for some analytical results. In this limiting case, the response of APC/C is no longer smooth but can only take on two values:

$$\left[\text{APC/C}\right]=\{\begin{array}{cc} 0 & \text{if}\;\;[\text{Cdk1}](t-\tau )<K\\ 1 & \text{if}\;\;[\text{Cdk1}](t-\tau )>K. \end{array}$$

**Fig 3:**
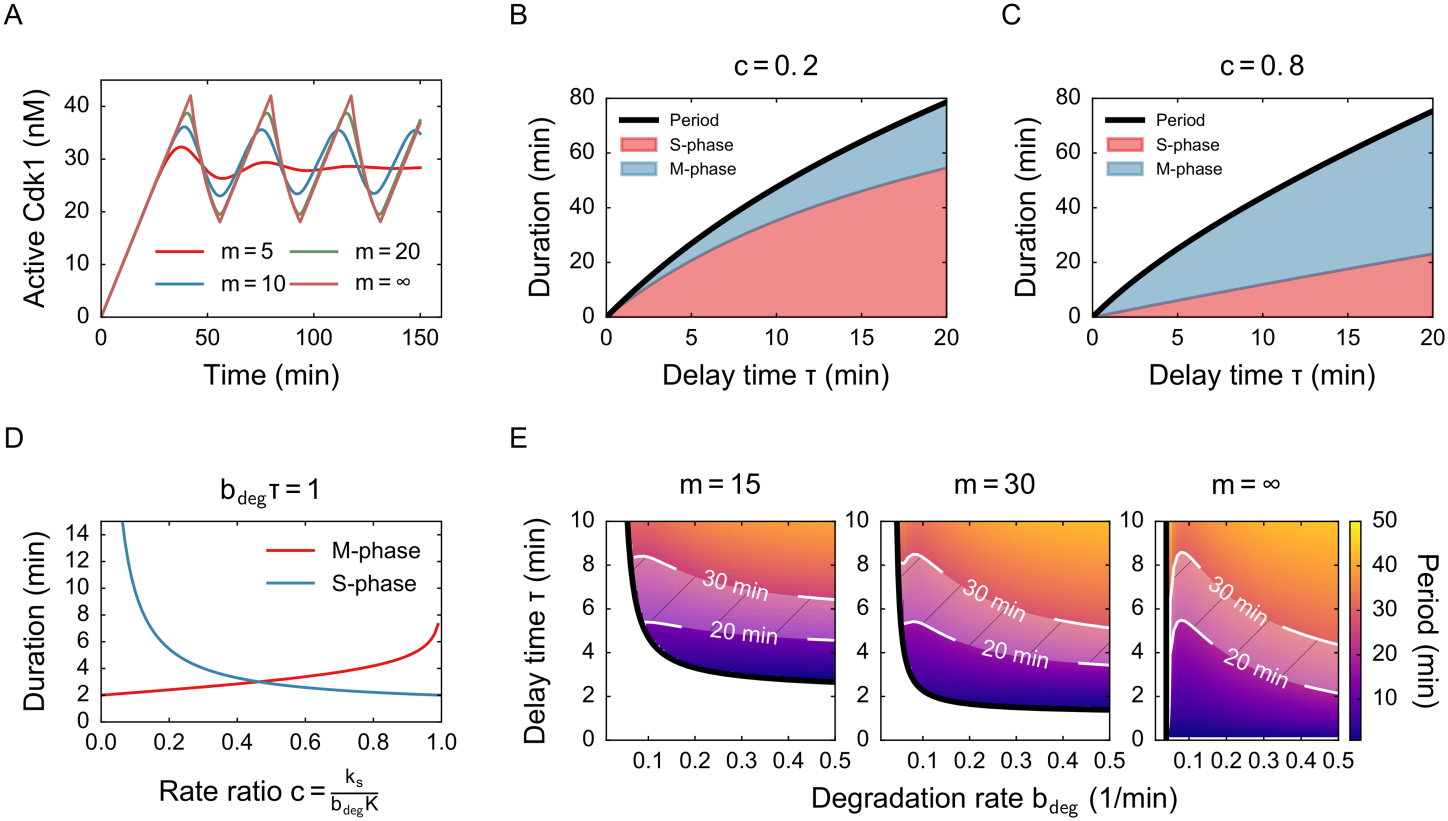
An approximation for high ultrasensitivity allows to study the period of the oscillations analytically. A) Time series for increasing values of *m* and for *m* = ∞. B) Duration of S phase and M phase for a low value of *c* as function of delay time. Other parameters: *k*_*s*_ = 0.64 nM/min, *b*_deg_ = 0.1 min^−1^ C) Duration of S phase and M phase for a high value of *c* as function of delay time. Other parameters: *k*_*s*_ = 2.28 nM/min, *b*_deg_ = 0.05 min^−1^ D) Duration of S phase and M phase as function of *c*. Equality of S and M phase duration is achieved roughly around *c* = 1/2. E) Period as function of *b*_deg_ and *τ* for *k*_*s*_ = 1.25 nM/min with realistic region indicated in white. The *m* dependence lies mostly in the existence of oscillations (the phase boundary, in black), but not in the period.

The equation can now be solved by hand (as in the book by Erneux [41], pp. 57-59), and it is possible to obtain explicit formulae for amplitude and period (see S1 Text). The period is given by the following expression:

$$P=\underset{\text{Degradation}\;\text{time}}{\underset{︸}{\tau +\frac{1}{b_{\text{deg}}}\text{ln}\left(1+\frac{c}{1-c}b_{\text{deg}}\tau \right)}}+\underset{\text{Accumulation}\;\text{time}}{\underset{︸}{\tau +\frac{1}{b_{\text{deg}}}\frac{1-c}{c}\left(1-e^{-b_{\text{deg}}\tau }\right)}},$$ (3)

where we have split the total period into the accumulation and degradation times, which are roughly the time of S phase and M phase. In both terms, the delay time *τ* appears explicitly. The period is dominated by a term 2*τ*, especially for delay times much larger than 1/*b*_deg_. The influence of the other terms depends on *c*: a very small *c* means accumulation is slow, which entails a longer S phase. Higher values of *c* (close to 1) correspond to longer M phase, which happens when the degradation rate is lower (Fig 3B and 3C).

After the first cycle, M phase and S phase duration are almost equal (Fig 2 in [20]). In our model this would correspond with a value of *c* of about 0.5 (Fig 3D and S4 Fig). Notably, this value of *c* is also the one for which oscillations are most likely to occur (Fig 2C). In the (*c*, *b*_deg_*τ*) phase diagram, the lowest point of the curve lies at *c* ≈ 1/2.

In embryos, the period is measured to be around 25 minutes (Fig 1A). Using this information, we can get an idea of realistic parameter ranges. Since *k*_*s*_ is relatively well constrained (1–1.5 nM/min) but *b*_deg_ and *τ* are more uncertain, we check what values of *b*_deg_ correspond with a period around 25 min (Fig 3E). The valid range for *b*_deg_ and *τ* depends only little on *m*. These pictures show that in any case *τ* is smaller than about 8 minutes and that a higher *τ* corresponds with lower *b*_deg_. A way to approximate *b*_deg_ is to assume that *c* = 1/2, since this is the value for which oscillations occur most easily and for which M and S phase have equal duration. Using the formula for *c* and substituting *c* = 1/2, *K* = 32 nM and *k*_*s*_ = 1.25 nM/min gives *b*_deg_ ≈ 0.08 min^−1^.

This simple model already illustrates some of the salient features of a time-delayed negative feedback system. Oscillations need a significant time delay *and* ultrasensitivity. The higher the ultrasensitivity, the less time delay is needed for the system to oscillate. The stability also depends on the ratio between accumulation and degradation rates. The period is largely independent of the exact value of *m*, which makes the formula we obtained analytically in the limiting case relevant. A main drawback of this model is that it uses one single delay value. In other words, the activity of APC/C depends on how active Cdk1 was exactly *τ* minutes ago. To relax this assumption, we now discuss how a more distributed delay affects the system dynamics.

### Systems with distributed time delays are less likely to oscillate

**Fig 4:**
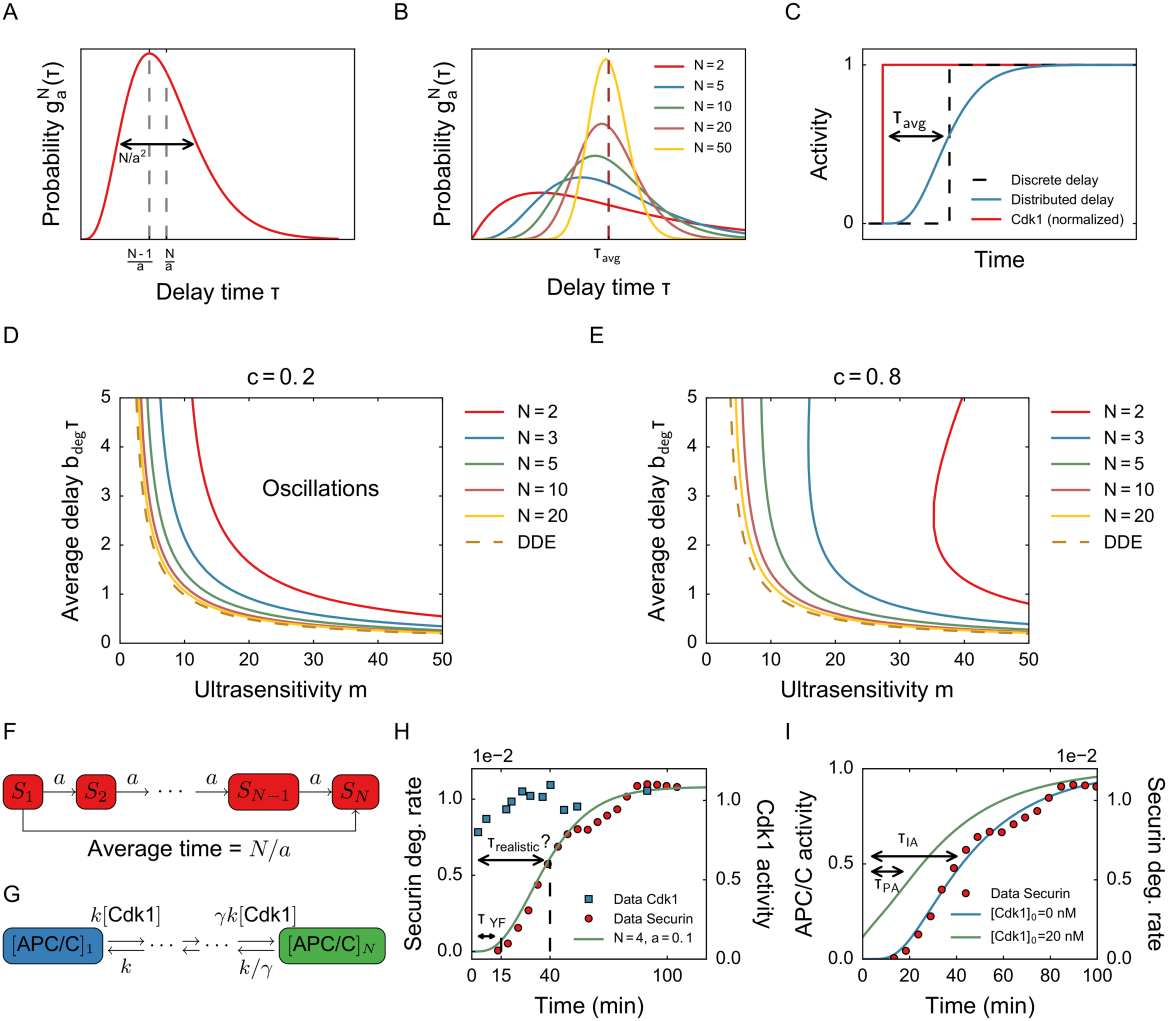
A distributed delay makes it less likely for the system to oscillate. A) Gamma distribution. The parameters *N* and *a* influence both width and position of the peak. B) When the average is fixed and *N* increases, the Gamma distribution becomes more peaked and converges to a Dirac delta distribution, in which the delay is a fixed value *τ*_avg_. C) Response of APC/C to a jump in Cdk1 activity for distributed delay. Compared with a model in which the delay is fixed, the response is much smoother. D) Phase diagram for low *c*. The region on the upper right is the region in which oscillations exist. E) Phase diagram for high *c*. The region on the upper right is the region in which oscillations exist. F) A linear chain of *N* reactions gives rise to a Gamma distribution. The number of steps and the rate of each step determine the average time delay. G) The model used by Yang and Ferrell [19] to model APC/C activation. Active Cdk1 catalyzes all steps in the cascade. The last step is made cooperative (parameter *γ*), in order to obtain an ultrasensitive response. H) When fitting a gamma distribution to the response of APC/C in the paper by Yang and Ferrell [19], we obtain an average delay of about 40 minutes. I) Using the model from Panel G but starting from a partially activated state, the resulting delay is much shorter.

A more realistic model is one which incorporates a delay distribution. An assumption in the previous model was that all APC/C molecules get activated at exactly the same time after Cdk1 molecules are activated. The molecular reactions that occur between these activations are highly stochastic, such that some APC/C molecules might be activated a bit earlier and some a bit later. To model this, we switch from a discrete delay time to a distributed delay. Although in principle any distribution can be used, we restrict ourselves here to the Gamma distribution. The Gamma distribution possesses some nice mathematical properties and is a reasonably accurate approximation of many delays occuring in nature [42]. It is characterized by two parameters, *N* and *a*, which influence the average and the width of the distribution (Fig 4A). In what follows, we choose the parameters *a* and *N* such that the average delay time is fixed. If we fix the average and let *N* increase, the distribution becomes more peaked (Fig 4B), and in the limit for *N* → ∞ the distribution reduces to one single value as studied in the previous sections.

Additional motivation for the use of Gamma distributions comes from a stochastic model for a chain of chemical reactions, in which a chemical species can be transformed into the next with a rate *a* (Fig 4F). In stochastic simulations of this system, the waiting time for one step is exponentially distributed with parameter *a*. The waiting time to go from the first to the last species then follows a Gamma distribution with parameters *a*, *N*. This observation can be generalized to more complicated reaction chains and has been used to speed up stochastic simulation of chemical reactions [43, 44]. The stochastic interpretion is merely a motivation for the use of this distribution, since we will keep all our models deterministic in this work. This motivation for the Gamma distribution has a clear analogue in the deterministic case, however. It can be shown (for example pp. 166-167 in [45] or pp. 123-126 in [42]) that a distributed delay equation with a Gamma distribution is equivalent to a system of ODEs, in which a chain of intermediate variables generates the time delay. The parameter *N* corresponds to the number of intermediate variables used (see Materials and methods). We fix an average delay time *τ*_avg_ while varying *N* in such a chain by choosing *a* = *N*/*τ*_avg_. These additional variables, or equivalently a distributed delay, lead to a smooth response of APC/C to a sudden change in Cdk1 activity (Fig 4C), as opposed to the discrete time delay in the previous sections. If the number of steps *N* becomes larger, and at the same time each step becomes faster, this system of equations will approximate a discrete delay system. This can be generalized to other linear systems [46] as there is always a distribution such that a linear system is equivalent to a distributed delay system. In a real biological system with intermediate species, the interactions will be likely nonlinear, following enzymatic kinetics, for example. In this case there is no formal equivalence with a distributed delay.

Using this distributed delay model, we again first checked the stability of the steady state and the region of existence of oscillations. Our analysis shows that the width of the distribution has a large influence on the regions of stability (Fig 4D and 4E). When *N* is small (a wide distribution), the steady state remains stable for a much larger range of parameter values than in the case of the discrete DDE. As *N* increases, the stability boundary eventually converges to the boundary for the discrete DDE case, as expected. For smaller *N*, there is a big difference depending on the ratio between accumulation and degradation rates (the parameter *c*). For higher *c*, oscillations only occur for very large values of *m*. The curve for *N* = 2 also clearly bends to the right, indicating that when the time delay increases, the steady state first loses stability and oscillations appear, but when the delay increases further the steady state regains stability. This behavior never occurs in the DDE model with a discrete delay. Note that for *N* = 1 (in which case the Gamma distribution is equal to an exponential distribution), oscillations are never possible. A distributed delay thus stabilizes the steady state and therefore suppresses oscillations. In the phase diagrams the curve for the DDE model lies to the left of the distributed delay curves, which means the region of oscillations is greatest in the DDE case. This general principle has also been observed in models of ecosystems [47], gene expression [48] and neuron dynamics [49]. This point is crucial: when modelers decide to include a time delay in a model, be it of a biological system or something else, the first choice is usually to use a discrete delay. Such a choice, however, biases the results towards instability and oscillations. A more realistic and conservative approach is to use a delay distribution such as the Gamma distribution. Although the width of the distribution clearly influences the stability and occurrence of oscillations, it has only a limited influence on the period (S5 Fig), which is mainly determined by the average time delay. The width does, however, influence the amplitude (S6 Fig).

We then set out to compare our model predictions to experimental measurements in the *Xenopus* embryonic cell cycle. Fig 4H shows the rate of securin degradation (a substrate of APC/C) in time in response to an initial increase in Cdk1 activity, as measured by Yang and Ferrell [19]. We fitted a Gamma distribution to these data and obtained values of *N* ≈ 4, *a* ≈ 0.1, which corresponds to an average delay of about 40 minutes, much more than what the delay would be if it was measured at the start of increasing APC/C activity (Fig 4H). This high value of *τ*_avg_ is not realistic to use in the delay model, especially given the fact that the period is only about 25 minutes. It is possible that this high measured value is due to the nature of the experiment, where the value of Cdk1 is switched from very low to very high, and APC/C starts completely from an inactive state. In the actual oscillating system, however, Cdk1 and APC/C fluctuate between states of low and high activity, but they do not necessarily “start from zero”. This is also illustrated by the analyzed time courses of cyclin B and Cdk1 activity in Fig 2 of [20], where cyclin B levels (and correspondingly, Cdk1 activity) oscillate between approx. 20nM and 40nM.

To illustrate that the nature of the experiment can alter the measured time delay, in Fig 4I, we show two simulations based on a cascade model which was also used by Yang and Ferrell [19]. This model describes APC/C activation as the result of a series of phosphorylation steps (Fig 4G), each of which is catalyzed by Cdk1-cyclin B (for model equations, see S1 Text). Although this model is not exactly equivalent to a distributed delay model, the response of APC/C to a sudden activation of Cdk1 is very similar. In addition, this model generates ultrasensitivity. If we suppose that the securin degradation rate data is generated by a jump in Cdk1 activity from 0 to 40 nM, we get a reasonable fit if we use *N* = 10 steps and *k* = 0.00675 min^−1^, corresponding to an average delay of *τ*_IA_ ≈ 40 min. Next, we run the same model, but now we start from a value of 20 nM instead of zero, which likely reflects more closely the actual system dynamics. This results in a much smaller measured delay value of *τ*_PA_ ≈ 20 min (Fig 4I), due to the fact that the intermediate states of APC/C are partially activated at the start. This observation illustrates that the delay obtained in an experiment is not necessarily the same as the delay time that needs to be used in a model. Additionally, it highlights the importance of how time delays are defined.

### A state-dependent delay models different activation and inactivation delays

**Fig 5:**
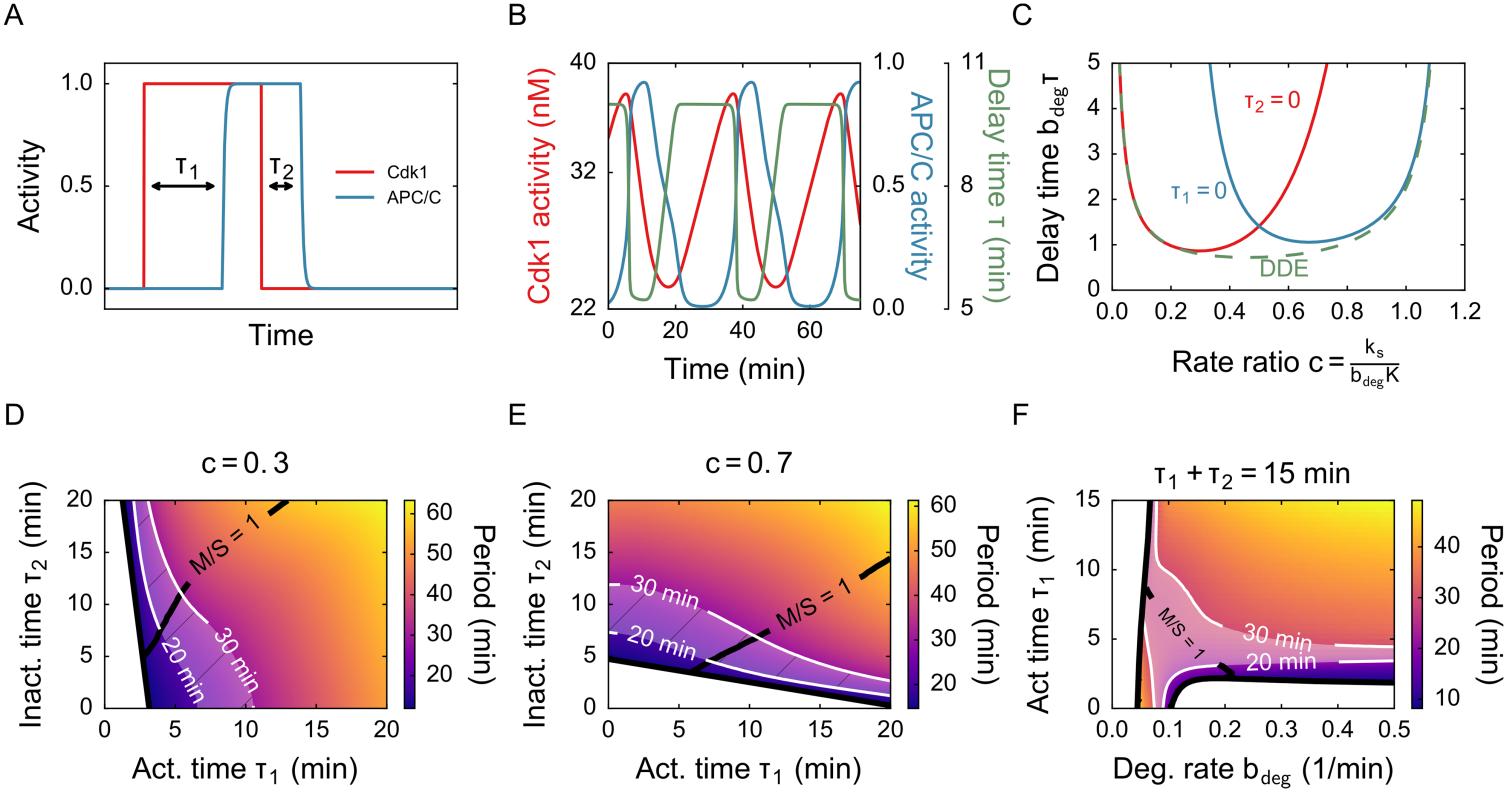
The importance of activation/inactivation delays depends on the accumulation and degradation rates. A) Response of APC/C to a sudden activation and inactivation of Cdk1. Two different time delays can be modeled using state-dependent delay equations. The response is smooth because APC/C is a separate variable, but still quite sharp if *β* is large. B) Time series for the model with state-dependent delay. The time delay switches rapidly between *τ*_1_ = 10 and *τ*_2_ = 5. Other parameters: *k*_*s*_ = 1 nM/min, *b*_deg_ = 0.1 min^−1^, *m* = 15, *p* = 5. C) Phase diagram when one of either *τ*_1_ or *τ*_2_ is zero. For very low and high values of *c* the phase boundary coincides with the boundary for the fixed delay model. D) Period as function of *τ*_1_ and *τ*_2_ for a low value of *c*. The indicated area denotes parameter values for which the period is between 20 and 30 minutes. The line denotes parameters where the M phase and S phase are equally long. Other parameters: *k*_*s*_ = 1.2 nM/min, *b*_deg_ = 0.125 min^−1^, *m* = 20, *β* = 5 min^−1^, *p* = 5. E) Same as D, but for a high value of *c*. Other parameters: *k*_*s*_ = 1 nM/min, *b*_deg_ = 0.0625 min^−1^, *m* = 20, *β* = 5 min^−1^, *p* = 5. F) Period as function of *b*_deg_ and *τ*_1_. The sum of *τ*_1_ and *τ*_2_ is fixed at 15 minutes and *k*_*s*_ = 1.25 nM/min, *m* = 20, *β* = 5 min^−1^, *p* = 5. The white area shows parameter values that give a realistic period, the line shows which values give an equal length of M and S phase. The results suggest that *b*_deg_ lies in the region around 0.1 min^−1^.

Fig 4I showed that a time delay can strongly depend on the current state of the system, i.e. the current activity of Cdk1 and APC/C. As during the actual cell cycle both Cdk1 and APC/C activities are continuously changing, it suggests that the effective time delay also changes accordingly. However, in the discrete and distributed delay models, APC/C always depends on Cdk1 in the same way. Another way to improve the relevance of a cell cycle model is, therefore, to include a state-dependent delay. Such delay could in principle change with each value of Cdk1 and APC/C activity, but here we focus on one potentially very large change of the delay time as the system oscillates. We ask ourselves what happens if the time delay *τ*_1_ in the *activation* of APC/C, measured by [19], is different from the time delay *τ*_2_ in *inactivation*, which hasn’t been measured yet (Fig 5A). One example of how such an asymmetry could arise is the following: imagine that APC/C is activated due to a series of phosphorylations by Cdk1-cyclin B as in the model used by Yang and Ferrell [19] (Fig 4G). Assuming that only the fully phosphorylated APC/C is active, the time delay for activation will be long as all sites need to be phosphorylated. However, inactivation can be quick when Cdk1 activity drops because only one site needs to be dephosphorylated in order to inactivate APC/C.

To address this issue of asymmetric time delays, we extend our model with a time delay *τ* that can depend on the state of the system. Systems with state-dependent delays are currently a subject of intense research, both from a purely mathematical point of view as for applications. State-dependent delays may arise naturally in a variety of systems, including drilling tools [50], laser dynamics [51] or economics [52]. They can arise in structured population models, where time to maturation depends on the population size. A lot of work in this context has been done on models of erythropoiesis (eg. [53, 54]). Furthermore state-dependent delays have also been used in models of disease spreading and predator-prey models [55]. From a mathematical point of view, state-dependent delays pose significant challenges, and results on stability and existence of solutions are fairly recent. For an overview of these issues, see [56].

We use a state-dependent delay to model the following phenomenon: when Cdk1 activity is going up, APC/C is activated with a time delay *τ*_1_. When APC/C activity is high, and Cdk1 activity drops due to cyclin degradation, APC/C activity will go down, but it reacts with a potentially different time delay *τ*_2_. The model equations we use are

$$\begin{array}{c} \begin{array}{cc} \frac{d[\text{Cdk1}]}{dt} & =k_{s}-b_{\text{deg}}\left[\text{Cdk1}\right]\left[\text{APC/C}\right]\\ \frac{d[\text{APC/C}]}{dt} & =\beta (\frac{{\left[\text{Cdk1}\right]}^{m}\left(t-\tau \right)}{K^{m}+{\left[\text{Cdk1}\right]}^{m}\left(t-\tau \right)}-\left[\text{APC/C}\right])\\ \tau ([\text{APC/C}]) & =\tau_{1}+\left(\tau_{2}-\tau_{1}\right)\frac{{\left[\text{APC/C}\right]}^{p}}{0.5^{p}+{\left[\text{APC/C}\right]}^{p}} \end{array} \end{array}$$ (4)

We included APC/C activity as a separate variable for purely technical reasons, as it is required to correctly implement the state-dependence. For large values of *β* and *τ*_1_ = *τ*_2_, this model approximates our first model (see S1 Text). The second change is that *τ* is now a function of APC/C: the time delay is close to *τ*_1_ when APC/C activity is low and close to *τ*_2_ when it is high. A Hill function is used to produce a smooth transition between these two regimes. Fig 5B shows an example time series, in which *τ* switches between a high and low value.

We first consider what happens in the most extreme case, namely when either activation (*τ*_1_) or inactivation (*τ*_2_) delay is zero. Imagine for instance that the inactivation time *τ*_2_ is zero, what should the activation delay *τ*_1_ then be such that the system oscillates? In this case, oscillations are possible for low *c* but become impossible for high *c* (Fig 5C). When instead the activation delay *τ*_1_ is zero, we have the inverse situation.

The value of *c* is, as we discussed, the ratio between accumulation and degradation rates. It also determines the value of the steady state: if *c* is high, the steady state of the system will have a higher APC/C activity (Fig 2F). We can deduce the following: if *c* is low, so steady state APC/C activity is low, it is easier to get oscillations with *τ*_2_ = 0 than it is with *τ*_1_ = 0. So in this case, the activation delay is important. If the steady state is high, *τ*_2_ is more important to go from a stable steady state to oscillations. These observations correspond to our intuition about the system and show that the state-dependent delay model is a meaningful extension. For low and high values of *c*, the phase diagram in Fig 5C coincides with the DDE diagram. This can be explained by noting that at the phase boundary, oscillation amplitude is small. If the oscillation then happens around a low steady state, APC/C activity will always stay low and therefore the time delay will stay around *τ*_1_. In this case the state-dependence is almost negligible and the system behaves like the discrete delay model with *τ* = *τ*_1_.

The influence of *τ*_1_ and *τ*_2_ on period and duration of M and S phase also depends on *c*. For smaller *c*, or stronger degradation, *τ*_1_ is most influential on period whereas for large *c*, or stronger accumulation, *τ*_2_ is most important (Fig 5D and 5E and S7 and S8 Figs). In order to have a period around 25 minutes and approximately equal S phase and M phase, it seems that *τ*_1_ and *τ*_2_ should sum to about 15 minutes. We can make another assessment of how large *b*_deg_ should be by fixing the sum of *τ*_1_ and *τ*_2_, fixing *k*_*s*_, and then plotting the period as function of *b*_deg_ and *τ*_1_ (Fig 5F and S9 Fig). This shows that *b*_deg_ should be around 0.1 min^−1^ in order to have oscillations of the right period. This value is biologically realistic (Table 1).

The state-dependent model mainly shows that the relative importance of activation and inactivation delay depends on the accumulation and degradation rates. Both of them contribute to the period of the oscillation. These observations will need to be taken into account when doing experiments to measure the time delay: if, for example, the activation delay measured is too high relative to the observed period, this could be compensated for by a lower inactivation delay or vice versa. Our model can give some constraints on the parameters, but experimental measurements will need to shed light on exactly in which parameter regime the cell cycle oscillations occur.

## Discussion

Our results show that a careful analysis of how to implement a time delay in a biological system can give new insights into realistic parameter ranges and underlying processes. From the most basic delay model, including one fixed value of the time delay, we conclude that a minimal delay value and ultrasensitivity are both needed to obtain oscillations. It is, however, mainly the delay time that dictates the period of the oscillations.

A more realistic model, with a distributed delay, shows that the discrete delay case is the most extreme one. Wider distributions actually stabilize an equilibrium, such that oscillations are harder to obtain. Whereas having a wider distribution strongly influences the stability of the oscillations, the period mostly depends on the average delay and only little on the variance. The distributed delay can be interpreted as resulting from a linear system with many steps. Although such a linear system is unrealistic, it gives an idea of how a time delay may be generated. In biological systems, a time delay usually originates in a lot of -possibly unknown- intermediate processes. A distributed delay is thus a natural choice in these cases, for using a discrete delay may lead to wrong conclusions [57]. This should be contrasted with some physical systems where the time delay is due to a response or signaling time. Those cases are better modeled by a discrete delay. A distributed delay can also be used to fit data and obtain an average delay out of it. Using this approach on data by Yang and Ferrell [19], we find a much larger time delay than the one the authors used in their model. This large delay may be due to the specific nature of the experiment, where the response is measured by changing Cdk1 activity from zero to active, whereas in the real oscillating system the delay may be rather associated to Cdk1 and APC/C getting activated from an intermediate level to an active state. Our initial simulations indeed show that in such a situation the effective time delay could be much shorter. An analysis of more biologically realistic models could shed light on such differences.

These findings also suggest that it might be necessary to move towards a more dynamic definition of a delay time, i.e. a delay that depends on the current activity of Cdk1 and APC/C. In order to address such a possibility, we studied a model with state-dependent delay. Experiments have only shown that there is a delay in the activation of APC/C. Using the state-dependent delay, we mimic what happens when the inactivation occurs with another time delay. In this model the relative importance of activation and inactivation times depends strongly on the ratio between accumulation and degradation rates ($c=\frac{k_{s}}{b_{\text{deg}}K}$). This parameter dictates whether the steady state of the system will have a low or high activity of APC/C. This in turn determines which delay should be large in order to escape from the steady state and obtain oscillations. We found that the oscillation period depends on both delays and the rate ratio *c*.

The question of how to model time delays is related to the problem of detecting them in time series. This is a nontrivial problem, both from an experimental and algorithmic point of view. We have illustrated in our estimation of the time delay from the data from Yang and Ferrell [19] that measuring a response in the system without feedback may not be appropriate as a measure for the time delay in the oscillating system. A more realistic estimate of the time delay in the feedback loop could be obtained from time series data of the oscillating activities of Cdk1-cyclin B and APC/C, but experimentally measuring these is complex. Different methods and algorithms exist for detecting time delays and reconstructing interactions from time series. There is a difference in methodology depending on whether the model is already known or not. If the model equations are known, and only the parameters need to be determined, the time delay can be fitted similarly as any other parameter. The literature on this subject is vast, and the fitting of parameters and an accurate quantification of the uncertainty is not obvious, especially when models become large [58–60]. The parameter fitting problem goes hand in hand with the issue of model selection: if different models accurately fit the data, which one is best? The problem can be even more difficult when the underlying equations are not known, and the goal is to infer the interactions from time series. The detection of time delays has received considerable interest, expecially in system exhibiting chaotic dynamics [61–63]. Extracting time delays between different interacting variables from such time series can be done in a variety of ways: both simple heuristics and sophisticated algorithms exist [64]. This is an important topic to study in order to bring the delay models closer to experiments.

Although we studied simple delay models of a particular biological system, the early embryonic cell cycle oscillator, many of our results are applicable to delay models in general. The models can be easily simulated on a computer (XPPAUT codes are included in S1 Text) and implemented for a wide range of biological oscillators. We hope that researchers who want to include a time delay in their biological models find inspiration in our results. Perhaps the most important take-away message is that implementing time delays differently can have a large impact on the resulting oscillations. Therefore care needs to be taken in both the choice of the model and the values of the relevant time delays.

## Materials and methods

### Experiments done for producing Fig 1

Female *Xenopus laevis* were primed using 100 units pregnant mare’s serum gonadotropin and induced using 500 units human chorionic gonadotropin. Eggs were collected 20 hours after induction by pelvic massage. The *in vitro* fertilization was performed by mixing eggs with smashed testes for 5 minutes and flooding with 0.1 × Marc’s Modified Ringer’s (MMR) buffer. The jelly coat of the fertilized embryos was removed 15 minutes after fertilization by treatment with 2% cysteine in 0.1 × MMR for 5 minutes. They were then washed three times with 0.5 × MMR. After collection, the parthenogenetically activated eggs were first treated for 5 minutes with 2% cysteine in 0.1 × MMR to remove the jelly coat, washed three times with 0.1 × MMR and subsequently activated by adding 0.5 μg/ml of calcium ionophore A23187 in 0.2 × MMR during 2 minutes. They were then washed three times with 0.5 × MMR. Both the fertilized and the parthenogenetically activated eggs were placed in an embryo chamber [65] at 24 °C in 0.5 × MMR and imaged using a Stemi 508 stereo microscope at a frame rate of 1 frame/30s.

Cycling extracts were prepared from *Xenopus* eggs according to the protocol by Murray [5]. They were supplemented with NLS-GFP (at a final concentration of ∼ 25 μM) and demembranated sperm nuclei (at a final concentration of ∼ 250 nuclei/(μl extract), and immediately taken from ice to room temperature. They were then loaded in Teflon tubes (Cole-Parmer PTFE, 06417-11) and imaged at 24 °C on a Leica TCS SPE confocal microscope.

### Equations for distributed delay

In order to model a distributed delay, we change [Cdk1](*t* − *τ*) for the expression

$$\int_{0}^{t}\left[\text{Cdk1}\right]\left(t-\tau \right)g\left(\tau \right)d\tau ,$$

where *g* is the distribution function. Where a discrete delay corresponds to a jump in the response, a distributed delay models a smoother time response (Fig 4B). This integral expression corresponds to the weighted average of all previous Cdk1 activities, weighted by the function *g*. The most likely delay time corresponds to the maximum of *g*, and the average delay time is given by $\int_{0}^{\infty }\tau g\left(\tau \right)d\tau$.

The density function of the Gamma distribution is given by

$$\begin{array}{c} g_{a}^{N}\left(\tau \right)=\frac{a^{N}}{(N-1)!}\tau^{N-1}e^{-a\tau }. \end{array}$$ (5)

The parameters *a* and *N* govern the average and the width of the distribution: The average is *N*/*a* and the variance is *N*/*a*^2^. The maximum of this function is obtained for *τ* = (*N* − 1)/*a*. In our models, we fix the average delay and vary *N*. Since *τ*_avg_ = *N*/*a*, this means *a* = *N*/*τ*_avg_.

The Gamma distribution has the mathematical property that a model with such a delay is exactly equivalent with a system of ordinary differential equations, where the number of equations needed is equal to the parameter *N*. In our case,

$$\begin{array}{c} \frac{d\left[\text{Cdk1}\right]}{dt}=k_{s}-b_{\text{deg}}\left[\text{Cdk1}\right]\frac{{\left(\int_{0}^{t}\left[\text{Cdk1}\right]\left(t-\tau \right)g_{N/\tau_{\text{avg}}}^{N}\left(\tau \right)\text{d}\tau \right)}^{m}}{K^{m}+{\left(\int_{0}^{t}\left[\text{Cdk1}\right]\left(t-\tau \right)g_{N/\tau_{\text{avg}}}^{N}\left(\tau \right)\text{d}\tau \right)}^{m}} \end{array}$$ (6)

is equivalent to

$$\begin{array}{c} \begin{array}{cc} \frac{d[\text{Cdk1}]}{dt} & =k_{s}-b_{\text{deg}}\left[\text{Cdk1}\right]\frac{y_{N}^{m}}{K^{m}+y_{N}^{m}}\\ \frac{dy_{1}}{dt} & =\frac{N}{\tau_{\text{avg}}}\left(\left[\text{Cdk1}\right]-y_{1}\right)\\ \frac{dy_{2}}{dt} & =\frac{N}{\tau_{\text{avg}}}\left(y_{1}-y_{2}\right)\\ & \vdots \\ \frac{dy_{N}}{dt} & =\frac{N}{\tau_{\text{avg}}}\left(y_{N-1}-y_{N}\right). \end{array} \end{array}$$ (7)

### Software

The discrete delay model was simulated using XPPAUT [66] and the pydelay package for Python [67]. The distributed delay model was converted into the equivalent ODE model (see Materials and methods), and then simulated using XPPAUT. The state-dependent delay model was also simulated using XPPAUT. The supplementary information contains XPP files for the three main models. Curves for phase diagrams were computed analytically and verified using DDE-BIFTOOL, a MATLAB/OCTAVE package for bifurcation analysis of delay equations [68]. The phase diagrams for the distributed delay were verified using the software AUTO contained in XPPAUT. The detection of period and duration of S and M phase was done using a custom Python algorithm, based on the extrema in the time series. As an approximation for S and M phase, we used the length of the increasing and decreasing parts of the time series during one period. The Python package XPPy [69] was used as an interface between XPPAUT and Python, for automating model runs and obtaining the timeseries. Data handling, formatting and plotting was done using Python. The color schemes used are based on the ones from Colorbrewer [70].

## Supporting information

S1 Fig A time delay destabilizes the steady state. The system settles into a constant, stable, steady state if the time delay is low (left). For increasing time delays, the system first exhibits damped oscillations (middle) and finally the steady state becomes unstable and sustained oscillations occur (right). The time delay at which the state becomes unstable depends on the parameters (Fig 2C and 2E).
(PDF)

S2 Fig Amplitude as function of *m* and *τ*. The amplitude of the oscillation (activity of Cdk1) as a function of *m* and *τ*. Compare with Fig 2E in the main text. Whereas the period jumps at the boundary, the amplitude increases gradually from 0 at the boundary to larger values farther away. The amplitude is influenced by *m* too, where the period depends almost solely on *τ*.
(PDF)

S3 Fig Amplitude as function of *k*_*s*_ and *b*_deg_. The amplitude of the oscillation (activity of Cdk1) as a function of *k*_*s*_ and *b*_deg_. Compare with Fig 2F in the main text. Whereas the period jumps at the boundary, the amplitude increases gradually from 0 at the boundary to larger values farther away.
(PDF)

S4 Fig Length of S phase and M phase as function of *c* and *τ*. In the main text (Fig 3D) we show the duration of S phase and M phase in the *m* → ∞ model. We concluded that they are equal for *c* ≈ 1/2. This picture shows that this holds too for the model with finite *m*.
(PDF)

S5 Fig Oscillation period in the distributed delay model. The period of the oscillation as function of *N* for different values of *m* and *τ*. Higher *N* corresponds to more peaked distributions. The period depends very little on *N* from a certain point onwards. The main influence on the period comes from *τ*. This figure supplements Fig 4 in the main text.
(PDF)

S6 Fig Oscillation amplitude in the distributed delay model. The amplitude of the oscillation as function of *N* for different values of *m* and *τ*. Higher *N* corresponds to more peaked distributions. In contrast to the period, the amplitude is influenced by all the parameters in this plot. This figure supplements Fig 4 in the main text.
(PDF)

S7 Fig Amplitude as function of *τ*_1_ and *τ*_2_ for low *c* in state-dependent delay model. Amplitude as function of *τ*_1_ and *τ*_2_, for low *c*. Corresponds to Fig 5D in the main text, which shows the period. The black line indicates where M and S phase have equal duration. Other parameters: *k*_*s*_ = 1.2 nM/min, *b*_deg_ = 0.125 min^−1^, *m* = 20, *β* = 5 min^−1^, *p* = 5.
(PDF)

S8 Fig Amplitude as function of *τ*_1_ and *τ*_2_ for high *c* in state-dependent delay model. Same as S7 Fig, but with a high value of *c*. Corresponds to Fig 5E in the main text. Other parameters: *k*_*s*_ = 1 nM/min, *b*_deg_ = 0.0625 min^−1^, *m* = 20, *β* = 5min^−1^, *p* = 5.
(PDF)

S9 Fig Amplitude as function of *b*_deg_ and *τ*_1_. Amplitude as function of *b*_deg_ and *τ*_1_. Corresponds to Fig 5F in the main text, which shows the period. The sum of *τ*_1_ and *τ*_2_ is fixed at 15 minutes and *k*_*s*_ = 1.25 nM/min, *m* = 20, *β* = 5 min^−1^, *p* = 5. The line shows which values give an equal length of M and S phase.
(PDF)

S1 Text Supplementary information. This file contains the mathematical analysis of the models and XPPAUT code for running model simulations.
(PDF)

## References

[1] Anderson GA, Gelens L, Baker J, Ferrell JE Jr. Desynchronizing Embryonic Cell Division Waves Reveals the Robustness of Xenopus Laevis Development. Cell Reports. 2017;21:37–46. doi: 10.1016/j.celrep.2017.09.017 28978482 10.1016/j.celrep.2017.09.017 PMC5679461

[2] Hara K, Tydeman P, Kirschner M. A Cytoplasmic Clock with the Same Period as the Division Cycle in Xenopus Eggs. Proceedings of the National Academy of Sciences. 1980;77(1):462–466. 10.1073/pnas.77.1.462 PMC348291 6928638

[3] Chang JB, Ferrell JE Jr. Mitotic Trigger Waves and the Spatial Coordination of the Xenopus Cell Cycle. Nature. 2013;500(7464):603–607. doi: 10.1038/nature12321 23863935 10.1038/nature12321 PMC3758429

[4] Gelens L, Anderson GA, Ferrell JE Jr. Spatial Trigger Waves: Positive Feedback Gets You a Long Way. Molecular Biology of the Cell. 2014;25(22):3486–3493. doi: 10.1091/mbc.E14-08-1306 25368427 10.1091/mbc.E14-08-1306 PMC4230609

[5] Murray AW. Cell Cycle Extracts. In: Methods in Cell Biology. vol. 36 of Xenopus laevis: Practical Uses in Cell and Molecular Biology; 1991 p. 581–605. 1839804

[6] Pines J. Cubism and the Cell Cycle: The Many Faces of the APC/C. Nature Reviews Molecular Cell Biology. 2011;12(7):427–438. doi: 10.1038/nrm3132 21633387 10.1038/nrm3132

[7] Bollen M, Gerlich DW, Lesage B. Mitotic Phosphatases: From Entry Guards to Exit Guides. Trends in Cell Biology. 2009;19(10):531–541. doi: 10.1016/j.tcb.2009.06.005 19734049 10.1016/j.tcb.2009.06.005

[8] Mochida S, Hunt T. Protein Phosphatases and Their Regulation in the Control of Mitosis. EMBO reports. 2012;13(3):197–203. doi: 10.1038/embor.2011.263 22482124 10.1038/embor.2011.263 PMC3323141

[9] Hoffmann I, Clarke PR, Marcote MJ, Karsenti E, Draetta G. Phosphorylation and Activation of Human Cdc25-C by Cdc2–Cyclin B and Its Involvement in the Self-Amplification of MPF at Mitosis. The EMBO Journal. 1993;12(1):53–63. 8428594 10.1002/j.1460-2075.1993.tb05631.x PMC413175

[10] McGowan CH, Russell P. Human Wee1 Kinase Inhibits Cell Division by Phosphorylating P34cdc2 Exclusively on Tyr15. The EMBO Journal. 1993;12(1):75–85. 8428596 10.1002/j.1460-2075.1993.tb05633.x PMC413177

[11] Tang Z, Coleman TR, Dunphy WG. Two Distinct Mechanisms for Negative Regulation of the Wee1 Protein Kinase. The EMBO Journal. 1993;12(9):3427–3436. 7504624 10.1002/j.1460-2075.1993.tb06017.x PMC413619

[12] Pomerening JR, Sontag ED, Ferrell JE Jr. Building a Cell Cycle Oscillator: Hysteresis and Bistability in the Activation of Cdc2. Nature Cell Biology. 2003;5(4):346–351. doi: 10.1038/ncb954 12629549 10.1038/ncb954

[13] Sha W, Moore J, Chen K, Lassaletta AD, Yi CS, Tyson JJ, et al Hysteresis Drives Cell-Cycle Transitions in Xenopus Laevis Egg Extracts. Proceedings of the National Academy of Sciences. 2003;100(3):975–980. doi: 10.1073/pnas.0235349100 10.1073/pnas.0235349100 PMC298711 12509509

[14] Heim A, Konietzny A, Mayer TU. Protein Phosphatase 1 Is Essential for Greatwall Inactivation at Mitotic Exit. EMBO reports. 2015;16(11):1501–1510. doi: 10.15252/embr.201540876 26396231 10.15252/embr.201540876 PMC4641502

[15] Mochida S, Maslen SL, Skehel M, Hunt T. Greatwall Phosphorylates an Inhibitor of Protein Phosphatase 2*A* That Is Essential for Mitosis. Science. 2010;330(6011):1670–1673. doi: 10.1126/science.1195689 21164013 10.1126/science.1195689

[16] Mochida S, Rata S, Hino H, Nagai T, Novák B. Two Bistable Switches Govern M Phase Entry. Current Biology. 2016;26(24):3361–3367. doi: 10.1016/j.cub.2016.10.022 27889260 10.1016/j.cub.2016.10.022 PMC5196020

[17] Kim SY, Ferrell JE Jr. Substrate Competition as a Source of Ultrasensitivity in the Inactivation of Wee1. Cell. 2007;128(6):1133–1145. doi: 10.1016/j.cell.2007.01.039 17382882 10.1016/j.cell.2007.01.039

[18] Trunnell NB, Poon AC, Kim SY, Ferrell JE Jr. Ultrasensitivity in the Regulation of Cdc25C by Cdk1. Molecular Cell. 2011;41(3):263–274. doi: 10.1016/j.molcel.2011.01.012 21292159 10.1016/j.molcel.2011.01.012 PMC3060667

[19] Yang Q, Ferrell JE Jr. The Cdk1–APC/C Cell Cycle Oscillator Circuit Functions as a Time-Delayed, Ultrasensitive Switch. Nature Cell Biology. 2013;15(5):519–525. doi: 10.1038/ncb2737 23624406 10.1038/ncb2737 PMC3728279

[20] Tsai TYC, Theriot JA, Ferrell JE Jr. Changes in Oscillatory Dynamics in the Cell Cycle of Early Xenopus Laevis Embryos. PLOS Biology. 2014;12(2):e1001788 doi: 10.1371/journal.pbio.1001788 24523664 10.1371/journal.pbio.1001788 PMC3921120

[21] Marlovits G, Tyson CJ, Novak B, Tyson JJ. Modeling M-Phase Control in Xenopus Oocyte Extracts: The Surveillance Mechanism for Unreplicated DNA. Biophysical Chemistry. 1998;72(1):169–184. doi: 10.1016/S0301-4622(98)00132-X 9652093 10.1016/s0301-4622(98)00132-x

[22] Mattingly HH., Sheintuch M, and Shvartsman SY. The Design Space of the Embryonic Cell Cycle Oscillator. Biophysical Journal. 2017;113(3):743–52. doi: 10.1016/j.bpj.2017.06.045 28793227 10.1016/j.bpj.2017.06.045 PMC5550316

[23] Novák B, Tyson JJ. Design Principles of Biochemical Oscillators. Nature Reviews Molecular Cell Biology. 2008;9(12):981–991. doi: 10.1038/nrm2530 18971947 10.1038/nrm2530 PMC2796343

[24] Ferrell JE Jr, Tsai TYC, Yang Q. Modeling the Cell Cycle: Why Do Certain Circuits Oscillate? Cell. 2011;144(6):874–885. doi: 10.1016/j.cell.2011.03.006 21414480 10.1016/j.cell.2011.03.006

[25] Félix MA, Labbé JC, Dorée M, Hunt T, Karsenti E. Triggering of Cyclin Degradation in Interphase Extracts of Amphibian Eggs by Cdc2 Kinase. Nature. 1990;346(6282):379–382. doi: 10.1038/346379a0 2142754 10.1038/346379a0

[26] Goldbeter A. A Minimal Cascade Model for the Mitotic Oscillator Involving Cyclin and Cdc2 Kinase. Proceedings of the National Academy of Sciences. 1991;88(20):9107–9111. doi: 10.1073/pnas.88.20.9107 10.1073/pnas.88.20.9107 PMC52661 1833774

[27] Novak B, Tyson JJ. Numerical Analysis of a Comprehensive Model of M-Phase Control in Xenopus Oocyte Extracts and Intact Embryos. Journal of Cell Science. 1993;106(4):1153–1168. 8126097 10.1242/jcs.106.4.1153

[28] Pomerening JR, Kim SY, Ferrell JE Jr. Systems-Level Dissection of the Cell-Cycle Oscillator: Bypassing Positive Feedback Produces Damped Oscillations. Cell. 2005;122(4):565–578. doi: 10.1016/j.cell.2005.06.016 16122424 10.1016/j.cell.2005.06.016

[29] Salazar C, Höfer T. Versatile Regulation of Multisite Protein Phosphorylation by the Order of Phosphate Processing and Protein–protein Interactions. FEBS Journal. 2007;274(4):1046–1061. doi: 10.1111/j.1742-4658.2007.05653.x 17257173 10.1111/j.1742-4658.2007.05653.x

[30] Salazar C, Höfer T. Multisite Protein Phosphorylation—from Molecular Mechanisms to Kinetic Models. FEBS Journal. 2009;276(12):3177–3198. doi: 10.1111/j.1742-4658.2009.07027.x 19438722 10.1111/j.1742-4658.2009.07027.x

[31] Srividhya J, Gopinathan MS. A Simple Time Delay Model for Eukaryotic Cell Cycle. Journal of Theoretical Biology. 2006;241(3):617–627. doi: 10.1016/j.jtbi.2005.12.020 16473373 10.1016/j.jtbi.2005.12.020

[32] Srividhya J, Gopinathan MS, Schnell S. The Effects of Time Delays in a Phosphorylation–dephosphorylation Pathway. Biophysical Chemistry. 2007;125(2–3):286–297. doi: 10.1016/j.bpc.2006.09.001 17014949 10.1016/j.bpc.2006.09.001

[33] Goodwin BC. Oscillatory behavior in enzymatic control processes. Advances in Enzyme Regulation. 1965;3:425–437. doi: 10.1016/0065-2571(65)90067-1 5861813 10.1016/0065-2571(65)90067-1

[34] Ruoff P, Vinsjevik M, Monnerjahn C, Rensing L. The Goodwin oscillator: on the importance of degradation reactions in the circadian clock. Journal of Biological Rhythms. 1999;14(6):469–479. doi: 10.1177/074873099129001037 10643743 10.1177/074873099129001037

[35] François P, Despierre N, Siggia ED. Adaptive Temperature Compensation in Circadian Oscillations. PLOS Computational Biology. 2012;8(7):e1002585 doi: 10.1371/journal.pcbi.1002585 22807663 10.1371/journal.pcbi.1002585 PMC3395600

[36] Lewis J. Autoinhibition with Transcriptional Delay: A Simple Mechanism for the Zebrafish Somitogenesis Oscillator. Current Biology. 2003;13(16):1398–1408. doi: 10.1016/S0960-9822(03)00534-7 12932323 10.1016/s0960-9822(03)00534-7

[37] Tyson JJ, Othmer HG. The dynamics of feedback control circuits in biochemical pathways. In: Progress in Theoretical Biology. vol. 5; 1978 p. 2–62.

[38] Monk NAM. Oscillatory Expression of Hes1, p53, and NF-*κ*B Driven by Transcriptional Time Delays. Current Biology. 2003;13(16):1409–1413. doi: 10.1016/S0960-9822(03)00494-9 12932324 10.1016/s0960-9822(03)00494-9

[39] Gonze D, Abou-Jaoudé W. The Goodwin Model: Behind the Hill Function. PLOS ONE. 2013;8(8):e69573 doi: 10.1371/journal.pone.0069573 23936338 10.1371/journal.pone.0069573 PMC3731313

[40] Cooke KL, Grossman Z. Discrete Delay, Distributed Delay and Stability Switches. Journal of Mathematical Analysis and Applications. 1982;86(2):592–627. doi: 10.1016/0022-247X(82)90243-8

[41] Erneux T. Applied Delay Differential Equations. Springer Science & Business Media; 2009.

[42] MacDonald N. Biological Delay Systems: Linear Stability Theory. Cambridge University Press; 1989.

[43] Barrio M, Leier A, Marquez-Lago TT. Reduction of Chemical Reaction Networks through Delay Distributions. The Journal of Chemical Physics. 2013;138(10):104114 doi: 10.1063/1.4793982 23514472 10.1063/1.4793982

[44] Leier A, Barrio M, Marquez-Lago TT. Exact Model Reduction with Delays: Closed-Form Distributions and Extensions to Fully Bi-Directional Monomolecular Reactions. Journal of The Royal Society Interface. 2014;11(95):20140108 doi: 10.1098/rsif.2014.0108 10.1098/rsif.2014.0108 PMC4006250 24694895

[45] Smith H. An Introduction to Delay Differential Equations with Applications to the Life Sciences. Springer Science & Business Media; 2010.

[46] Hinch R, Schnell S. Mechanism Equivalence in Enzyme–Substrate Reactions: Distributed Differential Delay in Enzyme Kinetics. Journal of Mathematical Chemistry. 2004;35(3):253–264. doi: 10.1023/B:JOMC.0000033258.42803.60

[47] Eurich CW, Thiel A, Fahse L. Distributed Delays Stabilize Ecological Feedback Systems. Physical Review Letters. 2005;94(15):158104 doi: 10.1103/PhysRevLett.94.158104 15904194 10.1103/PhysRevLett.94.158104

[48] Rateitschak K, Wolkenhauer O. Intracellular Delay Limits Cyclic Changes in Gene Expression. Mathematical Biosciences. 2007;205(2):163–179. doi: 10.1016/j.mbs.2006.08.010 17027040 10.1016/j.mbs.2006.08.010

[49] Meyer U, Shao J, Chakrabarty S, Brandt SF, Luksch H, Wessel R. Distributed Delays Stabilize Neural Feedback Systems. Biological Cybernetics. 2008;99(1):79 doi: 10.1007/s00422-008-0239-8 18523798 10.1007/s00422-008-0239-8

[50] Insperger T, Barton DAW, Stépán G. Criticality of Hopf Bifurcation in State-Dependent Delay Model of Turning Processes. International Journal of Non-Linear Mechanics. 2008;43(2):140–149. doi: 10.1016/j.ijnonlinmec.2007.11.002

[51] Martínez-Llinàs J, Porte X, Soriano MC, Colet P, Fischer I. Dynamical Properties Induced by State-Dependent Delays in Photonic Systems. Nature Communications. 2015;6:7425 doi: 10.1038/ncomms8425 26081000 10.1038/ncomms8425 PMC4557356

[52] Mackey MC. Commodity Price Fluctuations: Price Dependent Delays and Nonlinearities as Explanatory Factors. Journal of Economic Theory. 1989;48(2):497–509. doi: 10.1016/0022-0531(89)90039-2

[53] Mahaffy JM, Bélair J, Mackey MC. Hematopoietic Model with Moving Boundary Condition and State Dependent Delay: Applications in Erythropoiesis. Journal of Theoretical Biology. 1998;190(2):135–146. doi: 10.1006/jtbi.1997.0537 9538462 10.1006/jtbi.1997.0537

[54] Adimy M, Crauste F, Hbid M, Qesmi R. Stability and Hopf Bifurcation for a Cell Population Model with State-Dependent Delay. SIAM Journal on Applied Mathematics. 2010;70(5):1611–1633. doi: 10.1137/080742713

[55] Mitchell JL, Carr TW. Effect of State-Dependent Delay on a Weakly Damped Nonlinear Oscillator. Physical Review E. 2011;83(4):046110 doi: 10.1103/PhysRevE.83.046110 10.1103/PhysRevE.83.046110 21599243

[56] Hartung F, Krisztin T, Walther HO, Wu J. Chapter 5 Functional Differential Equations with State-Dependent Delays: Theory and Applications In: Handbook of Differential Equations: Ordinary Differential Equations. vol. 3 North-Holland; 2006 p. 435–545.

[57] Feng J, Sevier SA, Huang B, Jia D, Levine H. Modeling Delayed Processes in Biological Systems. Physical Review E. 2016;94(3):032408 doi: 10.1103/PhysRevE.94.032408 27739721 10.1103/PhysRevE.94.032408

[58] Lillacci G, Khammash M. Parameter Estimation and Model Selection in Computational Biology. PLOS Computational Biology. 2010;6(3):e1000696 doi: 10.1371/journal.pcbi.1000696 20221262 10.1371/journal.pcbi.1000696 PMC2832681

[59] Babtie AC, Stumpf MPH. How to deal with parameters for whole-cell modelling. Journal of The Royal Society Interface. 2017;14(133):20170237 doi: 10.1098/rsif.2017.0237 10.1098/rsif.2017.0237 PMC5582120 28768879

[60] Ashyraliyev M, Fomekong-Nanfack Y, Kaandorp JA, Blom JG. Systems biology: parameter estimation for biochemical models. FEBS Journal. 2009;276(4):886–902. doi: 10.1111/j.1742-4658.2008.06844.x 19215296 10.1111/j.1742-4658.2008.06844.x

[61] Hegger R, Bünner MJ, Kantz H, Giaquinta A. Identifying and Modeling Delay Feedback Systems. Physical Review Letters. 1998;81(3):558–561. doi: 10.1103/PhysRevLett.81.558

[62] Jüngling T, Soriano MC, Fischer I. Determining the sub-Lyapunov exponent of delay systems from time series. Physical Review E. 2015;91(6):062908 doi: 10.1103/PhysRevE.91.062908 10.1103/PhysRevE.91.062908 26172773

[63] Zhu S, Gan L. Incomplete phase-space method to reveal time delay from scalar time series. Physical Review E. 2016;94(5):052210 doi: 10.1103/PhysRevE.94.052210 27967148 10.1103/PhysRevE.94.052210

[64] Ma H, Leng S, Tao C, Ying X, Kurths J, Lai YC, et al Detection of time delays and directional interactions based on time series from complex dynamical systems. Physical Review E. 2017;96(1):012221 doi: 10.1103/PhysRevE.96.012221 29347206 10.1103/PhysRevE.96.012221

[65] Gelens L, Huang KC, Ferrell JE Jr. How Does the Xenopus Laevis Embryonic Cell Cycle Avoid Spatial Chaos? Cell Reports. 2015;12(5):892–900. doi: 10.1016/j.celrep.2015.06.070 26212326 10.1016/j.celrep.2015.06.070 PMC4531097

[66] Ermentrout B. Simulating, Analyzing, and Animating Dynamical Systems: A Guide to XPPAUT for Researchers and Students. SIAM, Philadelphia, USA; 2002.

[67] Flunkert V, Schoell E. Pydelay—a Python Tool for Solving Delay Differential Equations arXiv:09111633 [nlin]. 2009.

[68] Engelborghs K, Luzyanina T, Roose D. Numerical Bifurcation Analysis of Delay Differential Equations Using DDE-BIFTOOL. ACM Trans Math Softw. 2002;28(1):1–21. doi: 10.1145/513001.513002

[69] Nowacki J. XPPy; 2011.

[70] Brewer CA. ColorBrewer; 2013.

