# Supplementary figures and images for: Delay models for the early embryonic cell cycle oscillator
Source: PLoS One. 2018 Mar 26;13(3):e0194769. doi: 10.1371/journal.pone.0194769 (PMC5868829; doi:10.1371/journal.pone.0194769)

$\tau = 1$  min

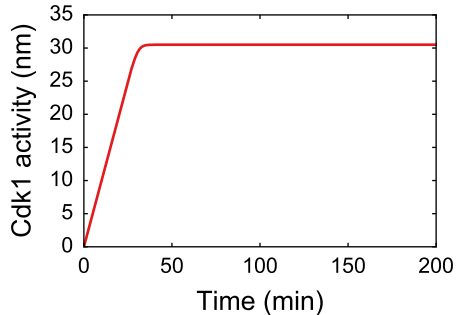

$\tau = 5$  min

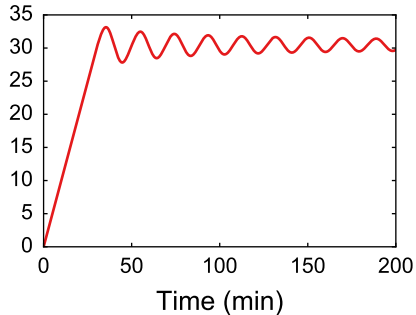

$\tau = 10$  min

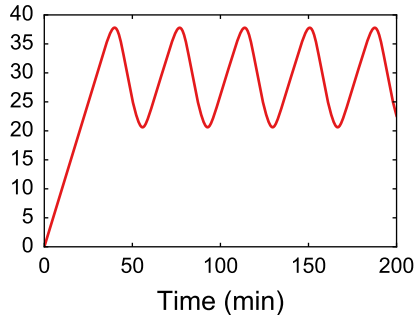

Supplement: S1 Fig — The system settles into a constant, stable, steady state if the time delay is low (left). For increasing time delays, the system first exhibits damped oscillations (middle) and finally the steady state becomes unstable and sustained oscillations occur (right). The time delay at which the state becomes unstable depends on the parameters (Fig 2C and 2E). (PDF) [file pone.0194769.s001.pdf]

$c = 0.4$

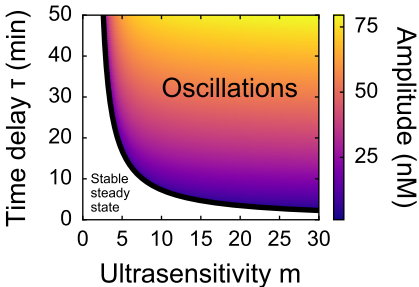

Supplement: S2 Fig — The amplitude of the oscillation (activity of Cdk1) as a function of m and τ. Compare with Fig 2E in the main text. Whereas the period jumps at the boundary, the amplitude increases gradually from 0 at the boundary to larger values farther away. The amplitude is influenced by m too, where the period depends almost solely on τ. (PDF) [file pone.0194769.s002.pdf]

$\tau = 10, m = 15$

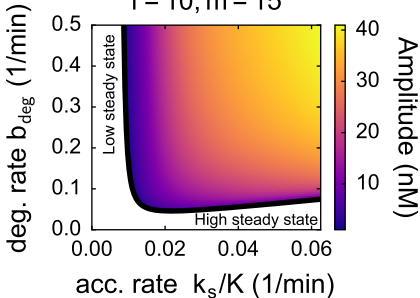

Supplement: S3 Fig — The amplitude of the oscillation (activity of Cdk1) as a function of ks and bdeg. Compare with Fig 2F in the main text. Whereas the period jumps at the boundary, the amplitude increases gradually from 0 at the boundary to larger values farther away. (PDF) [file pone.0194769.s003.pdf]

$m=10$

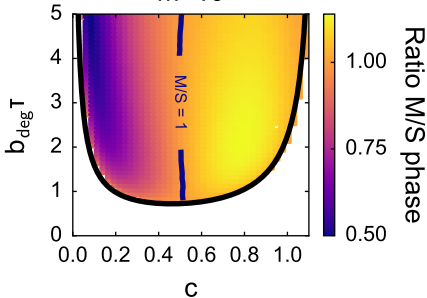

Supplement: S4 Fig — In the main text (Fig 3D) we show the duration of S phase and M phase in the m → ∞ model. We concluded that they are equal for c ≈ 1/2. This picture shows that this holds too for the model with finite m. (PDF) [file pone.0194769.s004.pdf]

$c = 0.2$

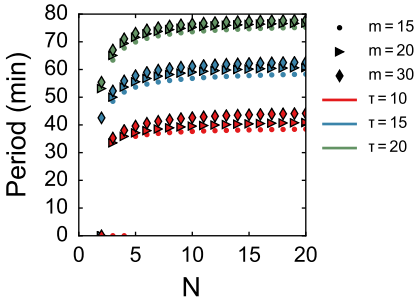

Supplement: S5 Fig — The period of the oscillation as function of N for different values of m and τ. Higher N corresponds to more peaked distributions. The period depends very little on N from a certain point onwards. The main influence on the period comes from τ. This figure supplements Fig 4 in the main text. (PDF) [file pone.0194769.s005.pdf]

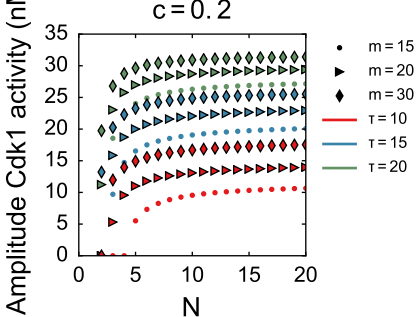

Supplement: S6 Fig — The amplitude of the oscillation as function of N for different values of m and τ. Higher N corresponds to more peaked distributions. In contrast to the period, the amplitude is influenced by all the parameters in this plot. This figure supplements Fig 4 in the main text. (PDF) [file pone.0194769.s006.pdf]

$c = 0.3$

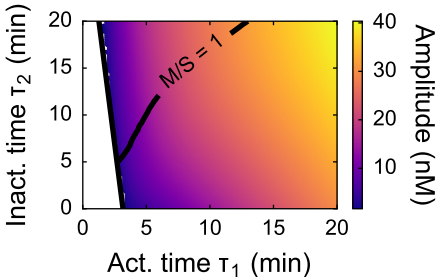

Supplement: S7 Fig — Amplitude as function of τ1 and τ2, for low c. Corresponds to Fig 5D in the main text, which shows the period. The black line indicates where M and S phase have equal duration. Other parameters: ks = 1.2 nM/min, bdeg = 0.125 min−1, m = 20, β = 5 min−1, p = 5. (PDF) [file pone.0194769.s007.pdf]

$c = 0.7$

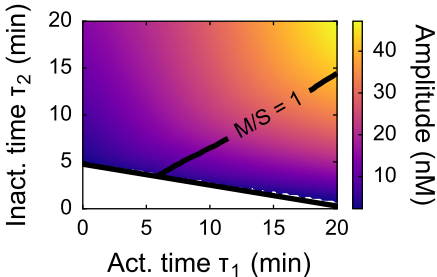

Supplement: S8 Fig — Same as S7 Fig, but with a high value of c. Corresponds to Fig 5E in the main text. Other parameters: ks = 1 nM/min, bdeg = 0.0625 min−1, m = 20, β = 5min−1, p = 5. (PDF) [file pone.0194769.s008.pdf]

$$\tau_1 + \tau_2 = 15 \text{ min}$$

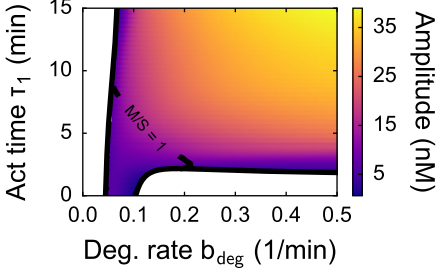

Supplement: S9 Fig — Amplitude as function of bdeg and τ1. Corresponds to Fig 5F in the main text, which shows the period. The sum of τ1 and τ2 is fixed at 15 minutes and ks = 1.25 nM/min, m = 20, β = 5 min−1, p = 5. The line shows which values give an equal length of M and S phase. (PDF) [file pone.0194769.s009.pdf]
