# Supplementary material for: Delay models for the early embryonic cell cycle oscillator
Source: PLoS One. 2018 Mar 26;13(3):e0194769. doi: 10.1371/journal.pone.0194769 (PMC5868829; doi:10.1371/journal.pone.0194769)
Supplement: S1 Text — This file contains the mathematical analysis of the models and XPPAUT code for running model simulations. (PDF) [file pone.0194769.s010.pdf]

Delay models for the early embryonic cell cycle oscillator
Supplementary Information

Jan Rombouts Alexandra Vandervelde Lendert Gelens

1 Mathematical analysis of the equation with one fixed delay

The Delay Differential Equation (DDE) equation reads (Eq. (1) in the main text)

$$\frac{d[\text{Cdk1}]}{dt} = k_s - b_{\text{deg}}[\text{Cdk1}]\frac{[\text{Cdk1}]^m(t-\tau)}{K^m + [\text{Cdk1}]^m(t-\tau)}. \tag{S1}$$

The meaning of the symbols in the equation:

Symbol	Meaning	Unit
[Cdk1]	Activity of Cdk1-Cyclin B complexes	nM
k_s	cyclin accumulation rate	nM / min
b_{deg}	Cyclin degradation rate	min^{-1}
m	Hill exponent	
K	Threshold value for APC/C activation	nM
τ	Delay time in APC/C activation	min

The activity of APC/C is not a separate variable, but is given by the delayed function of Cdk1:

$$[\text{APC/C}] = \frac{[\text{Cdk1}]^m(t-\tau)}{K^m + [\text{Cdk1}]^m(t-\tau)} \tag{S2}$$

To facilitate the mathematical analysis, we rescale the Cdk1 activity by K and the time by b_{deg}:

$$\begin{aligned} x &= \frac{[\text{Cdk1}]}{K} \\ c &= \frac{k_s}{b_{\text{deg}}K} \\ s &= b_{\text{deg}}t \\ \theta &= b_{\text{deg}}\tau, \end{aligned} \tag{S3}$$

such that we have the equation

$$\dot{x} = c - xf(x_\theta), \qquad f(x) = \frac{x^m}{1 + x^m}, \tag{S4}$$

where the dot means derivative with respect to s and $x_\theta = x(s - \theta)$.

1.1 Computing the fixed point/steady state of the system

The fixed point x^* of the equation satisfies $\dot{x} = 0$, or equivalently

$$f(x^*) = c/x^*. \tag{S5}$$

This equation has one unique solution, the value of which depends on c and m (See also Figure 2D). In the special case of $c = 1/2$, the fixed point $x^* = 1$. A formula for x^* can easily be found for $m = 1$, but for larger m we need to use numerical methods to find the fixed point.

1.2 Linearization and characteristic equation

In order to find out whether the fixed point is stable, we perform a linearization of the equation around the fixed point. Let $\xi = x - x^*$, assumed to be small. Then

$$\begin{aligned} \dot{\xi} &= \dot{x} \\ &= c - (x^* + \xi) f(x^* + \xi_\theta) \\ &\approx c - (x^* + \xi)(f(x^*) + f'(x^*)\xi_\theta) \\ &= -x^* f'(x^*)\xi_\theta - f(x^*)\xi, \end{aligned} \tag{S6}$$

where in the last line we used that $c - x^* f(x^*) = 0$ since x^* is a fixed point. To find out whether a small perturbation can grow in time, we substitute $\xi = e^{\lambda s}$ and calculate which values of λ satisfy the equation. If all solutions for λ have a negative real part, the perturbations die out and the solution is stable. This substitution leads to the equation

$$\lambda + f(x^*) + x^* f'(x^*) e^{\lambda\theta} = 0. \tag{S7}$$

This equation is transcendental (due to the exponential term), in contrast with characteristic equations for ODE systems, which are polynomial. Methods exist to determine conditions in which the real part of the solutions is negative (MacDonald, 1989). In this case, all real parts are negative until the time delay θ crosses a critical value. At this critical value, two of the solutions of this equation pass the imaginary axis and their real part becomes positive (Hopf bifurcation).

1.3 Calculation of the Hopf bifurcation and emergence of oscillations

At the Hopf bifurcation, we know that the solution must of of the form $\lambda = i\omega$. Substituting this and putting $f(x^*) = b, x^* f'(x^*) = d$, we obtain

$$i\omega + b + d(\cos\omega\theta - i\sin\omega\theta) = 0. \tag{S8}$$

Taking real and imaginary parts gives:

$$\begin{aligned} \omega &= d\sin\omega\theta \\ -b &= d\cos\omega\theta. \end{aligned} \tag{S9}$$

Rewriting this equations, we find

$$\begin{aligned} \omega &= \sqrt{d^2 - b^2} \\ \tan\omega\theta &= -\omega/b. \end{aligned} \tag{S10}$$

These equations are the basis for determining the phase boundaries (lines in Figure 2C, black boundaries in Figures 2E and 2F). If all parameters (such as $k_s, b_{\text{deg}}, \ldots$) are known, x^* can be computed and also $b = f(x^*)$ and $d = f'(x^*)$. The equations above can then be solved, first for ω and then for θ. The θ thus found is the critical value: delays higher than this value will destabilize the fixed point, and for these values oscillations exist. The whole curve separating oscillatory from stable steady state can also be determined parametrically, as is done in the book by Erneux (2009), for example. As an example we discuss Figure 2C but instead of τ, we use the scaled version θ. The figures in the main text were all generated by scaling back.

The boundary in the $c - \theta$ plane can be computed as follows. We use x^* as a parameter. For every value of x^*, we compute the associated c as $c = x^* f(x^*)$, and the associated θ using the formulas above:

$$\theta = \frac{1}{\sqrt{b^2 - d^2}} \arctan \frac{-\sqrt{b^2 - d^2}}{b}, \quad b = f(x^*), d = x^* f'(x^*). \tag{S11}$$

By plotting all these values in the plane, we obtain the phase boundary. For values of θ smaller than the critical one (regions below the curve), the steady state is stable. On the curve the Hopf bifurcation occurs and above it the system oscillates. This procedure can be repeated with other parameters too, as in Figures 2E and 2F.

These analytical curves can also be computed numerically, for example using the software DDE-BIFTOOL (Engelborghs et al., 2002; Sieber et al., 2014).

1.4 Analytical formula the period of the oscillation in the case $m \to \infty$

For the case $m \to \infty$ we can replace the Hill function by the much simpler expression

$$f(x) = \begin{cases} 0 & \text{if } x < 1 \\ 1 & \text{if } x \geq 1 \end{cases}. \tag{S12}$$

The delay equation then becomes

$$\dot{x} = \begin{cases} c & \text{if } x(t-\theta) < 1 \\ c - x & \text{if } x(t-\theta) \geq 1. \end{cases} \tag{S13}$$

It is possible to explicitly construct a periodic solution of this equation. We do this in the same style as Erneux (2009, pp. 57–59). The idea of the calculation will be made clear using following Figure.

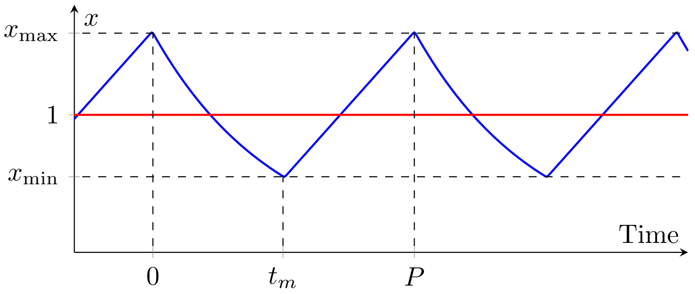

The periodic solution between $t = 0$ and $t = P$ (the period) consists of two parts: a decreasing part for $0 < t < t_m$ and an increasing part for $t_m < t < P$. By solving the equation on each part, we obtain

$$\begin{aligned} x_{\min} &= c + (1-c)e^{-\theta} \\ x_{\max} &= 1 + c\theta \\ t_m &= \ln\left(\frac{x_{\max} - c}{x_{\min} - c}\right) \\ P &= t_m + \frac{x_{\max} - x_{\min}}{c}. \end{aligned} \tag{S14}$$

Note that the periodic solution above only makes sense for $0 < c < 1$, which is also clear from the phase diagrams for $m \to \infty$. The formula given in the main text is obtained by substituting all formulas into the last one for P and then scaling time: $\theta = b_{\deg}\tau$, $P_{\text{unscaled}} = b_{\deg}P$.

2 Stability analysis for the equation with Gamma-distributed delay

The scaled equation with a distributed delay is

$$\dot{x} = c - xf(x_\theta), \tag{S15}$$

which is the same as the DDE, but now x_θ is not equal to $x(s-\theta)$ but

$$x_\theta = \int_0^s x(s-\theta)g(\theta)\mathrm{d}\theta. \tag{S16}$$

The function $g(\theta)$ is the distribution function, which in our case is the Gamma distribution. The fixed point of the equation and the linearization stay the same:

$$\dot{\xi} = -x^* f'(x^*)\xi_\theta - f(x^*)\xi, \quad \xi_\theta = \int_0^s \xi(s-\theta)g(\theta)\mathrm{d}\theta. \tag{S17}$$

When we now substitute $\xi = e^{\lambda s}$, the situation changes. The characteristic equation is

$$\lambda + b + d\int_0^\infty e^{-\lambda s}g(s)\mathrm{d}s = 0 \Leftrightarrow \lambda + b + d\hat{g}(\lambda) = 0. \tag{S18}$$

Note that we can change the upper limit from s to ∞ since $g(\theta) = 0$ for $\theta < 0$. Instead of a term $e^{-\lambda\theta}$, we now have the term

$$\hat{g}(\lambda) = \int_0^\infty e^{-\lambda s}g(s)\mathrm{d}s, \tag{S19}$$

which is the *Laplace transform* of the distribution g. In the case of a Gamma distribution with fixed mean τ_{avg} and parameter N, this can be computed (see any text on Laplace transforms) as

$$\int_0^\infty e^{-\lambda u}g^N_{N/\tau}(u)\mathrm{d}u = \frac{(N/\tau)^N}{(\lambda + (N/\tau))^N} = (1 + \frac{\lambda\tau}{N})^{-N}. \tag{S20}$$

This expression converges to $e^{-\lambda\tau_{\mathrm{avg}}}$ for $N \to \infty$. This corresponds to the fact that for $N \to \infty$, the distribution becomes more peaked and approaches a single delay value (Figure 4B). The characteristic equation now is

$$\lambda + b + d(1 + \frac{\lambda\tau}{N})^{-N} = 0, \tag{S21}$$

which can be rewritten as a polynomial equation in λ of degree $N+1$. The stability can now be computed by using criteria on the roots of polynomials (MacDonald, 1989). The fact that this is a polynomial equation reflects the equivalence between a Gamma distributed delay and a system of ODEs: a linear stability analysis of the ODE system (through characteristic polynomial of the Jacobian matrix) would lead to the same characteristic equation.

The phase boundaries can now be computed in a fashion analogous to the DDE case: substite $\lambda = i\omega$, separate real and imaginary parts, ...

Note that these phase boundaries can also be computed in AUTO (included in XPPAUT), since it is an ODE system. This is, in fact, how Figures 4D and 4E were created.

3 Yang and Ferrell's cascade model

For explaining why a time delay, as measured in an experiment, could depend on the initial value (the level of Cdk1 activity before activation), we run some simulations using the model by Yang and Ferrell (2013), which they explain in the supplementary information of their paper. Note that the parameter γ below corresponds with their parameter c. We used γ to avoid confusion with the rate ratio parameter c which we use.

The model is summarized by the following picture (cfr Figure 4G in the main text, which is a compressed version of the following)

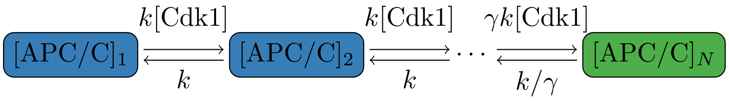

Here, $\mathrm{APC/C}_i$ represents APC/C which is phosphorylated $i-1$ times. The last one is the active form. The equations read

$$\begin{aligned}
\frac{d[\mathrm{APC/C}]_1}{dt} &= k[\mathrm{APC/C}]_2 - k[\mathrm{Cdk1}][\mathrm{APC/C}]_1 \\
\frac{d[\mathrm{APC/C}]_i}{dt} &= k[\mathrm{Cdk1}][\mathrm{APC/C}]_{i-1} + k[\mathrm{APC/C}]_{i+1} - k[\mathrm{Cdk1}][\mathrm{APC/C}]_i - k[\mathrm{APC/C}]_i \\
&\quad \text{for } i = 2 \cdots N-2 \\
\frac{d[\mathrm{APC/C}]_{N-1}}{dt} &= k[\mathrm{Cdk1}][\mathrm{APC/C}]_{N-2} + \frac{k}{\gamma}[\mathrm{APC/C}]_N - k\gamma[\mathrm{Cdk1}][\mathrm{APC/C}]_{N-1} - k[\mathrm{APC/C}]_{N-1} \\
\frac{d[\mathrm{APC/C}]_N}{dt} &= k\gamma[\mathrm{APC/C}]_{N-1} - \frac{k}{\gamma}[\mathrm{Cdk1}][\mathrm{APC/C}]_N
\end{aligned} \tag{S22}$$

The total amount of APC/C is conserved but is transformed between its different phosphorylation states. The last step is cooperative: $\gamma > 1$ means that for the last step, the forward rate is higher than the backwards rate. This is used to create an ultrasensitive response (we use $\gamma = 10$). For more details see the paper by Yang and Ferrell (2013) and their supplementary information.

4 State-dependendent delay

The equations for the model with a state-dependent delay are

$$\begin{aligned}
\frac{d[\mathrm{Cdk1}]}{dt} &= k_s - b_{\mathrm{deg}}[\mathrm{Cdk1}][\mathrm{APC/C}] \\
\frac{d[\mathrm{APC/C}]}{dt} &= \beta\left(\frac{[\mathrm{Cdk1}]^m(t-\tau)}{K^m + [\mathrm{Cdk1}]^m(t-\tau)} - [\mathrm{APC/C}]\right) \\
\tau([\mathrm{APC/C}]) &= \tau_1 + (\tau_2 - \tau_1)\frac{[\mathrm{APC/C}]^p}{0.5^p + [\mathrm{APC/C}]^p}
\end{aligned} \tag{S23}$$

The function τ is a Hill function, used to model a smooth transition between a higher and lower delay time which depends on APC/C activity.

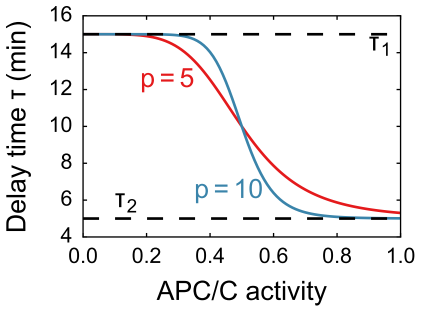

If β is very large and τ is constant, this system approximates the original 1 equation model. This corresponds to doing a quasi-steady state assumption on the APC/C variable.

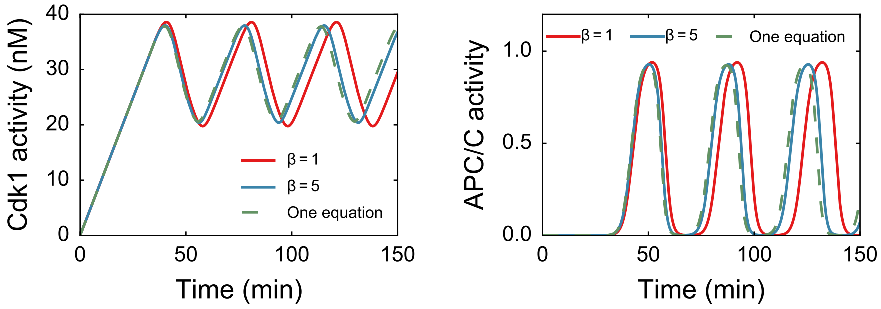

Using the same scaling as before $x = [\mathrm{Cdk1}]/K$, $s = b_{\mathrm{deg}}\tau$ gives us

$$\begin{aligned} \dot{x} &= c - xy \\ \dot{y} &= \tilde{\beta}\big(f(x(s-\theta(y))) - y\big), \end{aligned} \tag{S24}$$

where $c = k_s/K/b_{\mathrm{deg}}$, $\tilde{\beta} = \beta/b_{\mathrm{deg}}$, $\theta(y) = b_{\mathrm{deg}}\tau(y))$ and $f(x) = x^m/(1+x^m)$ and for ease of notation we set $y = [\mathrm{APC/C}]$.

The fixed point of these equations satisfies $c = xf(x), y = f(x)$, which corresponds exactly with the fixed point for the original model.

4.1 Linear stability analysis

Linearizing around x^*, y^* gives the equations

$$\begin{aligned} \dot{\xi} &= -x^*\eta - y^*\xi \\ \dot{\eta} &= \tilde{\beta}(f'(x^*)\xi_{\theta^*} - \eta), \end{aligned} \tag{S25}$$

where $\xi = x - x^*$, $\eta = y - y^*$ and $\theta^* = \theta(y^*)$, the delay time evaluated at the fixed point. This linearization (using a constant delay, which is the state-dependent delay evaluated at the fixed point) is the correct one (Hartung et al., 2006).

The characteristic equation is

$$\lambda^2 + (y^* + \tilde{\beta})\lambda + \tilde{\beta}y^* + \tilde{\beta}x^* f'(x^*)e^{-\lambda\theta^*} \tag{S26}$$

It is interesting to note that in the limit of $\tilde{\beta} \to \infty$, this reduces to the characteristic equation for one DDE.

As before, the phase boundary can be computed by inserting $\lambda = i\omega$ to find the Hopf bifurcation and solving for the relevant parameters. The analysis is a bit more involved now but completely follows (MacDonald, pp. 89-90).

Note that where before we obtained a critical delay time parameter for oscillations to occur, in this model we obtain a critical steady-state delay time θ_c^*. From this, we need to solve for θ_1 and θ_2 by using (recall that $\theta = b_{\text{deg}}\tau$, the scaled version of the delay time).

$$\theta^* = \theta_1 + (\theta_2 - \theta_1)\frac{y^{*p}}{0.5^p + y^{*p}}. \tag{S27}$$

When all parameters except the delay times are fixed, θ^* can be computed analytically and the equation above gives a relation between θ_1 and θ_2. This relation is linear, and corresponds to the straight line which is the phase boundary in Figures 5D and 5E.

These analytical curves can also be computed numerically using the software DDE-BIFTOOL (Engelborghs et al., 2002; Sieber et al., 2014).

5 Numerical codes

Below we provide the different numerical codes used to analyze the various delay models. The software XPPAUT (Ermentrout, 2002) can be downloaded from `http://www.math.pitt.edu/~bard/xpp/xpp.html`

5.1 Discrete delay

```
#simple delayed ultrasensitive oscillator

hill(x)=x^m/(K^m+x^m)

#x is Cdk1 activity

x'=ks-bdeg*x*hill(delay(x, tau))

x(0)=0
init x=0
```

```
par ks=1, bdeg=0.1, m=15, tau=10, K=32

#y denotes apc, the delayed function of Cdk1.

aux y=hill(delay(x, tau))


@ delay=100, total=200

#set some sliders for changing parameters
@ s1=tau, slo1=0, shi1=50, s2=ks, slo2=0, shi2=2, s3=m, slo3=0, shi3=50
done
```

5.2 Distributed delay equation

The following code is used to simulate the system of ODEs which is equivalent to the equation with a Gamma distributed delay. The number of variables N has to be changed in the file itself.

```
#N, the parameter of the distribution that determines the width,
#corresponds to the number of intermediate variables.
#N needs to be changed at 4 positions in this file.

#Hill function definition
hill(x)=x^m/(K^m+x^m)

#x0 is the Cdk1 variable
x0'=ks - bdeg*x0*hill(x10)

#The intermediate variables. The parameter a is N/tau to obtain an
#average delay of tau
%[1..10]
x[j]'=10/tau*(x[j-1]-x[j])
%

par tau=10, ks=1, m=15, K=32, bdeg=0.1

#APC/C is the delayed function of Cdk1, and the last intermediate variable
#represents the delayed Cdk1.
aux y=hill(x10)

@ delay=100, total=200

#set some sliders for changing parameters
@ s1=tau, slo1=0, shi1=50, s2=ks, slo2=0, shi2=2, s3=m, slo3=0, shi3=50

done
```

5.3 State-dependent delay

```
#State-dependent delay

hill(x)=x^m/(K^m+x^m)

#x is Cdk1, y is APC/C

x'=ks-bdeg*x*y
y'=beta*(hill(delay(x,tau(y))) - y)

#tau switches between tau1 and tau2, smoothness depends on exponent p
tau(y)=tau1 + (tau2-tau1)*y^p/(0.5^p + y^p)

#auxilary variable for plotting tau
aux tau=tau(y)

par tau1=10, tau2=10, m=15, ks=1, bdeg=0.1, K=32, beta=5, p=5

@ total=200, delay=100
@ s1=tau1, slo1=0, shi1=10, s2=tau2, slo2=0, shi2=10, s3=bdeg, slo3=0, shi3=0.5
done
```

References

Engelborghs, K., Luzyanina, T., and Roose, D. (2002). Numerical Bifurcation Analysis of Delay Differential Equations Using DDE-BIFTOOL. *ACM Trans. Math. Softw.*, 28(1):1–21.

Ermentrout, B. (2002). *Simulating, Analyzing, and Animating Dynamical Systems: A Guide to XPPAUT for Researchers and Students.* SIAM, Philadelphia, USA.

Erneux, T. (2009). *Applied Delay Differential Equations.* Springer Science & Business Media.

Hartung, F., Krisztin, T., Walther, H.-O., and Wu, J. (2006). Chapter 5 Functional Differential Equations with State-Dependent Delays: Theory and Applications. In *Handbook of Differential Equations: Ordinary Differential Equations*, volume 3, pages 435–545. North-Holland.

MacDonald, N. (1989). *Biological Delay Systems: Linear Stability Theory.* Cambridge University Press.

Sieber, J., Engelborghs, K., Luzyanina, T., Samaey, G., and Roose, D. (2014). DDE-BIFTOOL Manual - Bifurcation analysis of delay differential equations. *arXiv:1406.7144 [math].*

Yang, Q. and Ferrell, J. E. J. (2013). The Cdk1–APC/C cell cycle oscillator circuit functions as a time-delayed, ultrasensitive switch. *Nature Cell Biology*, 15(5):519–525.
